# LTβR-RelB signaling in intestinal epithelial cells protects from chemotherapy-induced mucosal damage

**DOI:** 10.3389/fimmu.2024.1388496

**Published:** 2024-05-30

**Authors:** Qiangxing Chen, Amanda R. Muñoz, Anna A. Korchagina, Yajun Shou, Jensine Vallecer, Austin W. Todd, Sergey A. Shein, Alexei V. Tumanov, Ekaterina Koroleva

**Affiliations:** ^1^ Department of Microbiology, Immunology and Molecular Genetics, University of Texas Health Science Center at San Antonio, San Antonio, TX, United States; ^2^ Department of Gastroenterology, Second Xiangya Hospital, and Research Center of Digestive Disease, Central South University, Changsha, Hunan, China

**Keywords:** LTβR, LIGHT, lymphotoxin, RelB, IL-22, methotrexate, intestinal damage

## Abstract

The intricate immune mechanisms governing mucosal healing following intestinal damage induced by cytotoxic drugs remain poorly understood. The goal of this study was to investigate the role of lymphotoxin beta receptor (LTβR) signaling in chemotherapy-induced intestinal damage. LTβR deficient mice exhibited heightened body weight loss, exacerbated intestinal pathology, increased proinflammatory cytokine expression, reduced IL-22 expression, and proliferation of intestinal epithelial cells following methotrexate (MTX) treatment. Furthermore, LTβR^-/-^IL-22^-/-^ mice succumbed to MTX treatment, suggesting that LTβR- and IL-22- dependent pathways jointly promote mucosal repair. Although both LTβR ligands LIGHT and LTβ were upregulated in the intestine early after MTX treatment, LIGHT^-/-^ mice, but not LTβ^-/-^ mice, displayed exacerbated disease. Further, we revealed the critical role of T cells in mucosal repair as T cell-deficient mice failed to upregulate intestinal LIGHT expression and exhibited increased body weight loss and intestinal pathology. Analysis of mice with conditional inactivation of LTβR revealed that LTβR signaling in intestinal epithelial cells, but not in Lgr5^+^ intestinal stem cells, macrophages or dendritic cells was critical for mucosal repair. Furthermore, inactivation of the non-canonical NF-kB pathway member RelB in intestinal epithelial cells promoted MTX-induced disease. Based on these results, we propose a model wherein LIGHT produced by T cells activates LTβR-RelB signaling in intestinal epithelial cells to facilitate mucosal repair following chemotherapy treatment.

## Introduction

Chemotherapy-induced intestinal damage poses a pervasive challenge, affecting up to 90% of patients undergoing chemotherapeutic treatments (1–3). The severity of this issue varies based on factors such as disease type, progression, drug type, and dosing regimen. The resultant gastrointestinal injury manifests in distressing symptoms like nausea, vomiting, diarrhea, and pain (2). Patient-specific risk factors, including age, ethnicity and gender also contribute to the varying susceptibility to intestinal damage during chemotherapy (4, 5). Beyond the immediate physical toll, chemotherapy-induced intestinal damage significantly impacts the quality of life for affected individuals (6). Moreover, it can compromise the effectiveness of treatments, leading to worse clinical outcomes, and potential economic repercussions due to the increased cost of care. Strikingly, reports indicate that 7.5% of deaths in chemotherapy patients result from nonselective toxicity rather than the disease itself (7). Therefore, therapeutic approaches such as combination of drug therapies and fecal microbiota transplantation are being developed to prevent or alleviate intestinal mucositis (8–12). However, despite these efforts, therapeutic targets remain limited, highlighting the need for a deeper understanding of the immune mechanisms governing mucosal repair following chemotherapy.

Due to rapid turnover of intestinal epithelial cells (IEC), the gastrointestinal (GI) tract is particularly sensitive to antineoplastic drugs such as methotrexate (MTX) and 5-Fluorouracil (5-FU) which inhibit cell growth and division (1, 13). MTX is a structural analog of folic acid which prevents folate metabolism via competitive inhibition of dihydrofolate reductase, resulting in the suppression of *de novo* synthesis of purines and pyrimidines (14). 5-FU mainly suppresses the action of thymidylate synthase but can also induce direct cytotoxicity through incorporation of its products into RNA and DNA (15). Animal models of chemotherapy-induced mucositis utilizing MTX and 5-FU treatments have been developed (2, 16, 17). Although the role of proinflammatory cytokines such as TNF, IL-6, IL-1 and reactive oxygen species (ROS) in pathogenesis of chemotherapy-induced mucositis is well recognized (1, 18), the immune mechanisms controlling the mucosal repair remain poorly understood.

IL-22 is an important cytokine of the interleukin-10 (IL-10) family of cytokines, produced by several hematopoietic cells, including helper T (Th) cells and innate lymphoid cells (ILCs) (19–22). IL-22 signals through the IL-22 receptor (IL-22R) paired with the IL-10Rβ subunit (23, 24). IL-10Rβ is ubiquitously expressed while IL-22R is selectively expressed by IECs and is involved in the regulation of epithelial repair and innate immunity (22, 25, 26). Furthermore, IL-22 can act on epithelial cells to induce secretion of antimicrobial proteins Reg3β and Reg3γ, which have been proposed to suppress inflammation and promote tissue recovery (27, 28). Additionally, IL-22 was shown to act directly on mouse and human intestinal stem cells (ISCs) to induce activation of the signal transducer and activator of transcription 3 (STAT3) to drive ISCs proliferation to increase organoid formation *in vitro* (26, 29). Moreover, a previous study revealed that group 3 ILCs (ILC3s) safeguard ISCs through production of IL-22 after MTX-induced acute small intestinal damage (30). However, a recent study suggested that ILC3-driven IEC proliferation in response to MTX-induced epithelial injury is independent of IL-22 (31). Furthermore, several studies demonstrated that IL-22 can exacerbate disease in psoriasis (32) and in several models of intestinal inflammation (33–36). Therefore, further understanding of IL-22-dependent and IL-22-independent pathways contributing to mucosal repair following chemotherapy-induced intestinal damage is critical for developing effective therapies.

Lymphotoxin beta receptor (LTβR), a core member of the tumor necrosis factor (TNF) receptor superfamily, exhibits wide expression across non-lymphocyte populations, including epithelial cells, dendritic cells (DCs), macrophages, mast cells, and stromal cells (37–39). LTβR interacts with two ligands: heterotrimeric lymphotoxin (LTα1β2, or LT) and homotrimeric LIGHT (TNFSF14), which are primarily expressed by lymphocytes and ILCs (37, 40). LTβR signaling serves pleiotropic functions, which include the control of lymphoid organ development and maintenance, as well as the regulation of inflammation and protective immunity to infections (38, 41). LTβR signaling activates canonical as well as non-canonical NF-κB signaling pathways to mediate both pro-inflammatory and anti-inflammatory responses (39, 42). Several studies have highlighted the protective role of LTβR signaling, which promotes mucosal healing in chemically-induced and infectious colitis models (43–47). Intriguingly, previous studies revealed the critical role of LTβR signaling in controlling IL-22 production by ILC3s in response to the mucosal bacterial pathogen *Citrobacter rodentium* (47) as well as in the DSS colitis model (45). Considering the role of ILC3s and IL-22 in MTX-induced mucosal repair (26, 30), we hypothesized that LTβR-dependent regulation of ILC3s and IL-22 mediates protection against chemotherapy-induced intestinal damage.

The goal of this study was to investigate the role of LTβR signaling in chemotherapy-induced intestinal damage using animal models of disease. Our data suggest that LIGHT-expressing T cells interact with LTβR on intestinal epithelial cells to induce non-canonical NF-κB signaling for protection against MTX-induced intestinal damage. Moreover, we show that LTβR and IL-22 pathways jointly protect from MTX-induced injury. Additionally, LTβR signaling also protects against 5-FU induced epithelial damage. These results support a novel role of LTβR signaling in mucosal repair following chemotherapy-induced intestinal injury by controlling cooperation of T cells and intestinal epithelial cells.

## Materials and methods

### Mice

All animal studies were conducted in accordance with the University of Texas Health Science Center at San Antonio Institutional Animal Care and Use Committee. 8–14 week old male and female mice were used for experiments. Age and sex matched littermate controls were used for all experiments. C57BL/6 (wild-type, WT) mice, RORγt^-/-^ (48), TCRβδ^-/-^ (49), IL-22^-/-^ (50), RORγt-Cre (48), Villin-Cre, Jax #021504 (51), LysM-Cre (52), CD11c-Cre (53), and Lgr5-EGFP-IRES-CreERT2 mice (54) (all on C57BL/6 background) were purchased from the Jackson Laboratory (Bar Harbor) and bred at the University of Texas Health Science Center at San Antonio. LTβR floxed (45), RelB floxed (55), LTβR^-/-^ (45), LTβ^-/-^ (56) and LIGHT^-/-^ (TNFSF14^-/-^) (57) mice were described previously. RORγt-LTβ^-/-^ mice were generated by crossing LTβ floxed mice (58) with RORγt-Cre transgenic mice (48). Vil-LTβR^-/-^, CD11c-LTβR^-/-^, LysM-LTβR^-/-^ and Lgr5-LTβR^-/-^ mice were generated by crossing LTβR floxed mice with CD11c-Cre (53), LysM-Cre (52), and Lgr5-EGFP-IRES-CreERT2 mice (54), respectively. Lgr5-EGFP-IRES-CreERT2 (54) mice were intercrossed with LTβR^-/-^ mice to generate Lgr5-reporter mice on LTβR-deficient background. To induce Cre-recombination, these mice were treated with 5 mg of tamoxifen for 4 consecutive days by oral gavage. Efficiency of *ltb*, *ltbr*, *relb* targeted gene deletion was validated in previous publications (43, 45, 47, 55, 59). All mice used in this research were housed under specific-pathogen-free conditions in line with National Institutes of Health guidelines.

### Intestinal damage models

For MTX-induced intestinal damage, 8–14 week old mice were treated *i.p.* with 120 mg/kg of Methotrexate (MTX, RPI) on day 0 and 60 mg/kg on day 1. Mice were euthanized and tissues collected on day 2 or 5. For survival studies, mice were weighed daily and euthanized on day 14 or if body weight loss reached 20%. For 5-FU induced colitis, 8–12 week old mice were treated *i.p.* with 50 mg/kg of 5-fluorouracil (5-FU, Sigma-Aldrich) on days 0, 1, 2, 3. Mice were euthanized on day 5 and small intestine, cecum and colon were removed for analysis.

### Assessment of 5-FU-induced colitis

The disease score was determined as an average of body weight loss (0 points, no weight loss; 1 point, weight loss of 1 to 5%; 2 points, weight loss of 5 to 10%; 3 points, weight loss of 10 to 20%; 4 points, weight loss >20%), signs of rectal bleeding (0 points, no blood in feces; 1point, positive hemoccult test; 2 points, dark feces; 3 points, visible blood in feces or traces of blood near anus; 4 points, gross bleeding from anus) and stool consistency (0 points, well-formed pellet; 1 point, soft pellet; 2 points, loose stool; 3 points, diarrhea; 4 points, no stool with dehydration). The scores were added to obtain a disease score ranging from 0 (healthy) to 16 (maximal activity of the disease). If the cecum was included, the cecum appearance score was determined as 0 points (normal), 1 point (slightly abnormal size), 2 points (significantly abnormal size) and 3 points (abnormal size with blood).

### Histology

Small intestines, cecums and colons were dissected from mice and fixed in 10% neutral buffered formalin. Paraffin-embedded tissue sections were stained with hematoxylin and eosin (H&E) for tissue pathology evaluation. Images were taken with the Keyence BZ-X800 microscope. Small intestine pathology was scored as previously described (60). Villus, epithelium, inflammation, infiltration, crypt length and abscess, and bleeding, were evaluated on the scale from 0 to 3 and scores were summarized: villus length (0 = normal, 1 = short, 2 = extremely short), villus tops (0 = normal, 1 = damaged, 2 = severely damaged), epithelium (0 = normal, 1 = flattened, 2 = damaged, 3 = severely damaged), inflammation (0 = no infiltration, 1 = mild infiltration, 2 = severe infiltration), crypts (0 = normal, 1 = mild crypt loss, 2 = severe crypt loss), crypt abscesses (0 = none, 1 = present) and bleeding (0 = none, 1 = present). For cecum and colon histopathology score, we used a previously described scoring system (61).

### Immunohistochemistry

5-Bromo-2′-deoxyuridine (BrdU, BD Biosciences, 100 mg/kg) was injected *i.p.* to mice two hours prior to analysis. Small intestines were fixed in 10% neutral buffered formalin and paraffin embedded. Sections were deparaffinized, rehydrated, and treated with 2 M HCl for 30 min at 37°C, and washed 3 times with PBS for 5 minutes, followed by 0.5% Triton X-100 for 30 minutes at room temperature. Tissue sections were blocked with goat serum at 37°C for 30 minutes and incubated with anti-BrdU antibody (Biolegend, clone 3D4) at 1:50 dilution at 4°C overnight. Sections were then incubated with HRP-conjugated goat anti-mouse IgG antibody (Biolegend) at 1:200 dilution at 37°C for 1h. Tissue sections were developed using DAB (Biolegend) and counterstained with hematoxylin. BrdU-positive cells were counted in 4 to 8 crypts per section. For Alcian Blue and Nuclear Fast Red staining slides were deparaffinized using Xylene and hydrated to distilled water. Slides were then incubated in 3% acetic acid for 3 min, stained in Alcian Blue solution pH 2.5 (American MasterTech) for 45 min, washed in running tap water, counter stained in nuclear fast red solution (American MasterTech) for 5 min, washed in running tap water, dehydrated to 100% ethanol, cleared in xylene, and mounted with Cytoseal 60 (Thermo Scientific) mounting medium. Images were taken with the Keyence BZ-X800 microscope.

### RNA isolation and real-time reverse transcription PCR analysis

RNA from tissue or cultured cells was extracted using E.Z.N.A. Total RNA Kit I (Omega Bio-tek). RNA from lamina propria and intraepithelial fraction was isolated using RNeasy Micro Kit (QIAGEN). cDNA synthesis and real-time PCR were performed as described previously (43) using Power SYBR Green master mix (Applied Biosystems). Relative mRNA expression of target genes was determined using the comparative 2-^ΔΔCt^ method and normalized to HPRT. Primers used are listed in **Supplementary Table 1**.

### Epithelial cell line CMT-93

CMT-93 cells (mouse rectal carcinoma cell line, ATCC) were cultured in DMEM (Corning) containing 10% FBS. Cells were treated with medium containing 5 μM MTX, or 0.5 μg/ml of agonistic αLTβR antibody (ACH6 clone, provided by Biogen Idec). Cells were incubated for 24 h before being harvested for RNA isolation.

### Preparation of epithelial cells, intraepithelial lymphocytes, and lamina propria cells

To isolate epithelial cells, intestines were opened longitudinally, washed, cut, and incubated in DMEM supplemented with 5% FBS, antibiotics and 1mM DTT at 37°C with rotation (170 rpm) for 20 minutes and vortexed for 30 sec. Pieces were then incubated for additional 20 minutes with rotation (37°C) in PBS/15mM EDTA. Crypts were further digested with serum free DMEM with 2 mg/ml of Collagenase D (Roche) for 30 minutes with rotation (37°C). EC suspensions were passed through 70 µm cell strainer, resuspended in complete media and overlaid on the top of a 20%:40% Percoll (GE Healthcare) gradient. Epithelial cells were collected at the interphase of the 20%:40% Percoll gradient, washed and resuspended in DMEM. Intraepithelial lymphocytes (IELs) and lamina propria (LP) lymphocytes were isolated as described previously (62). Briefly, the small intestines were removed, opened longitudinally, and washed in cold PBS to remove fecal material. The whole small intestine or the ileum were cut in 1 cm pieces and incubated in RPMI 1640 media supplemented with 3% FBS, 15mM HEPES, 1 mM penicillin-streptomycin, and 2 mM EDTA with shaking at 150 rpm for 20 min at 37°C to remove epithelium and IEL. IELs were collected in the supernatants and passed through a mesh screen and separated by 40%:80% Percoll gradient. For LP isolation, the remaining tissues were digested in serum-free RPMI media containing 200 μg/ml Liberase TM (Roche) and 0.05% DNAse I (Sigma) on a shaker for 40 min at 37°C. The digested tissue was passed through a mesh strainer, washed with RPMI media containing 3% FBS and separated by a 40%:80% Percoll gradient.

### Flow cytometry

For flow cytometry analysis, IELs and LP were preincubated for 20 min with anti-CD16/32 Fc-blocking mAb (2.4G2) and Zombie NIR™ Fixable viability dye (Biolegend) prior to surface staining. For cell surface staining single cell suspensions were incubated on ice with conjugated antibodies in PBS containing 2% of FBS. The following antibodies were used for surface staining: anti-MHCII (M5/114.15.2), anti-CCR2 (475301), anti-CD45 (30-F11), anti-CD8a (53–6.7), anti-NK1.1 (PK136), anti-CD11b (M1/70), anti-CD11c (N418), anti-TCRβ (H57–597), anti-Ly6G (1A8), anti-CD64 (X54–5/7.1), anti-Siglec-F (S17007L), anti-B220 (RA3–6B2), anti-CD4 (GK1.5), anti-CD3 (17A2), anti-CD8b (YTS156.7.7), anti-CD25 (PC61). For the transcriptional factors staining the following antibodies were used: anti-Foxp3 (MF-14) and anti-RORγt (Q31–378). For intracellular staining, cells were fixed and permeabilized with True-Nuclear™ transcriptional factor buffer set (Biolegend) according to the manufacturer’s protocol. For Lgr5-GFP reporter staining, the following antibodies were used: anti-EpCAM (G8.8), anti-TER-119 (TER-119), anti-CD117 (c-Kit) (2B8), anti-CD31 (MEC13.3). All antibodies were purchased from BD Biosciences or Biolegend. Samples were acquired using an FACSCelesta or Cytek Aurora (Cytek Biosciences), and data were analyzed using FlowJo 10 software.

### Statistical analysis

All statistics were determined using GraphPad Prism software (v9). Statistical significance was determined using one-way ANOVA or two-way ANOVA with Tukey’s multiple comparison test, Mann-Whitney test, Kruskal Wallis test with Dunn’s correction, or unpaired Student’s t-test, as appropriate. Survival was assessed using the Log-rank (Mantel-Cox) and Gehan-Breslow-Wilcoxon tests. Not significant, p > 0.05 (ns); p< 0.05 (*); p< 0.01 (**); p< 0.001 (***); p< 0.0001 (****).

## Results

### LTβR signaling protects from chemotherapy-induced intestinal damage

**Figure 1:**
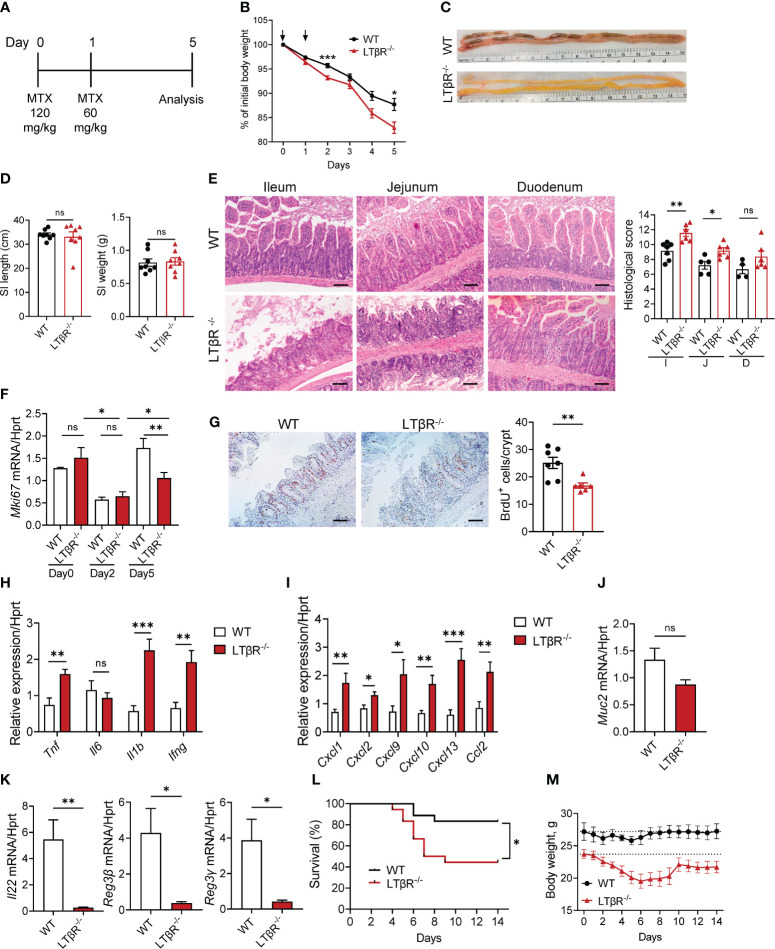
LTβR signaling protects against MTX-induced intestinal damage. **(A)** Schematic of the experiment. WT and LTβR^-/-^ mice were injected i.p. with MTX on day 0 (120 mg/kg) and day 1 (60 mg/kg), and small intestine (SI) collected at day 5. **(B)** Body weight change. Black arrows: days of MTX treatment. n=25–28 mice per group. **(C)** Representative photographs of SI. **(D)** Measurements of SI. **(E)** Representative H&E images and histopathology scores. Scale bars, 100μm. I, Ileum; J, Jejunum; D, Duodenum. **(F)** Ki-67 mRNA expression in ileum at indicated time points. n=4–7 mice per group. **(G)** Representative images of BrdU^+^ cells/crypt in the ileum. Scale bars, 100μm. **(H–K)** Expression of cytokines **(H)**, chemokines **(I)**, Muc2 **(J)**, and IL-22 and antimicrobial proteins **(K)** in the ileum and quantification of BrdU^+^ cells measured by real-time PCR. n=7–8 mice per group. **(L, M)** Survival analysis (n=18 mice per group, **L**) and long-term body weight analysis (n=5 mice per group, dotted lines represent median starting body weight in each group, **M**). **(C–E)** Data represents 1 out of 6 independent experiments with similar results. (**B**, **F–L)** Data is combined from 2–6 independent experiments with similar results. Data shown as mean ± SEM. Statistics were determined using two-way ANOVA with Geisser-Greenhouse correction **(B, M)**, unpaired t test **(D–K)**, and Log-rank (Mantel-Cox) test **(L)**. ns, not significant; *p<0.05, **p<0.01, ***p<0.001.

LTβR signaling is a known regulator of intestinal inflammation (43–45, 63, 64). To investigate the role of LTβR signaling in chemotherapy-induced intestinal damage, we employed an acute epithelial injury model induced by MTX (1, 2) (**Figure 1A**). Compared to WT mice, LTβR^-/-^ mice exhibited increased weight loss (**Figures 1B, M**) and increased mortality (**Figure 1L**) after MTX treatment. Macroscopic examination of small intestines on day 5 revealed severe pathology in LTβR^-/-^ mice compared to control mice (**Figure 1C**) while the length and weight of the small intestines remained unchanged (**Figure 1D**). Histological analysis revealed severe destruction of the epithelial layer in LTβR^-/-^ mice characterized by shortened villi, inflammatory cell infiltration, and increased loss of crypts (**Figure 1E**). Consistently, histopathology scores were significantly increased in the ileum and jejunum of LTβR^-/-^ mice, with the duodenum exhibiting less pronounced damage (**Figure 1E**). Crypt regenerative capacity was reduced in both WT and LTβR^-/-^ mice at day 2 after MTX administration (**Figure 1F**). While epithelial cell proliferation, measured by Ki-67 expression and BrdU incorporation, remained reduced in LTβR^-/-^ mice, it was restored in WT mice by day 5 after MTX administration (**Figures 1F, G**).

Expression of key proinflammatory cytokines TNF, IL-1β and IFNγ, but not IL-6 was upregulated in the ileum of LTβR^-/-^ mice at day 5 after MTX treatment (**Figure 1H**), as well as expression of chemokines CXCL1, CXCL2, CXCL9, CXCL10, CXCL13, and CCL2 (**Figure 1I**). Expression of IL-22 was significantly downregulated in the ileum of LTβR^-/-^ mice compared to WT controls (**Figure 1K**). Expression of IL-22 dependent antimicrobial proteins Reg3β and Reg3γ in the ileum of LTβR^-/-^ mice was also reduced compared to WT mice (**Figure 1K**). LTβR signaling is known to promote goblet cell differentiation and production of mucins in the gut during *Listeria monocytogenes* infection or DSS-induced colitis (45, 59). Interestingly, we did not detect significant reduction of Muc2 expression in the gut of LTβR^-/-^ mice (**Figure 1J**), suggesting that other LTβR-dependent factors contribute to mucosal repair after MTX-induced injury. Collectively, these data indicate that LTβR signaling is essential for the intestinal repair and control of inflammation after MTX-induced injury.

5-FU is another commonly used chemotherapeutic agent employed in the therapy of various cancers, which can cause damage to intestinal epithelial cells and result in intestinal mucositis (15, 65, 66). To test the role of LTβR signaling in a 5-FU model of chemotherapy-induced intestinal injury, we treated WT and LTβR^-/-^ mice with 50 mg/kg 5-FU daily for 4 days (**Supplementary Figure S1**). 5-FU treated LTβR^-/-^ mice exhibited aggravated body weight loss (**Supplementary Figure S1A**), increased clinical disease score (**Supplementary Figure S1B**) and shortening of the colon (**Supplementary Figure S1C**). Histological analysis of colon and cecum sections of 5-FU treated LTβR^-/-^ mice revealed severe mucosal damage characterized by loss of goblet cells and decreased crypt density which was accompanied by mass immune cell infiltration (**Supplementary Figure S1D**). Expression of proinflammatory cytokines TNF, IL-6, IL-1β and IFNγ was upregulated in the colon of 5-FU treated LTβR^-/-^ mice compared to control mice, whereas IL-22 levels were similar (**Supplementary Figure S1E**). These data indicate that LTβR signaling also contributes to intestinal protection in 5-FU chemotherapy-induced intestinal inflammation.

### LTβR signaling controls accumulation of B cells, neutrophils, CD8αα^+^ and CD4^+^ T cells in the small intestine early after MTX treatment

To define immune cell types in the small intestine early after MTX administration, we compared SI intraepithelial lymphocytes (IELs) and lamina propria (LP) immune cells in WT mice at steady state and at day 2 after MTX administration by flow cytometry. Gating strategy is shown on **Supplementary Figure S2**. We found an increased frequency of T cells (CD3^+^) and non-conventional CD8αα^+^ T cells in the IEL fraction after MTX administration (**Supplementary Figure S3A**). Interestingly, in the LP, frequency of Tregs was increased, although we did not find increased frequency of CD3^+^ T cells (**Supplementary Figure S3B**). Analysis of myeloid cell populations revealed increased frequency of macrophages and neutrophils (**Supplementary Figure S3C**) after MTX administration. Gene expression analysis revealed rapid induction of proinflammatory cytokines TNF, IL-6, IL-1β and IFNγ, as well as IL-22 at day 2 (**Supplementary Figure S3D**). In contrast, by day 5 after MTX administration, expression of these cytokines returned to steady state levels (**Supplementary Figure S3D**). Expression of IFNγ-induced chemokines CXCL9, CXCL10 (67), neutrophil-recruiting chemokines CXCL1, CXCL2 (68), and CXCL13 and CCL2 chemokines was upregulated on day 2 after MTX administration and reduced to baseline by day 5 (**Supplementary Figure S3E**). These data indicate that MTX rapidly induces inflammation and promotes immune cell infiltration into the small intestine.

**Figure 2:**
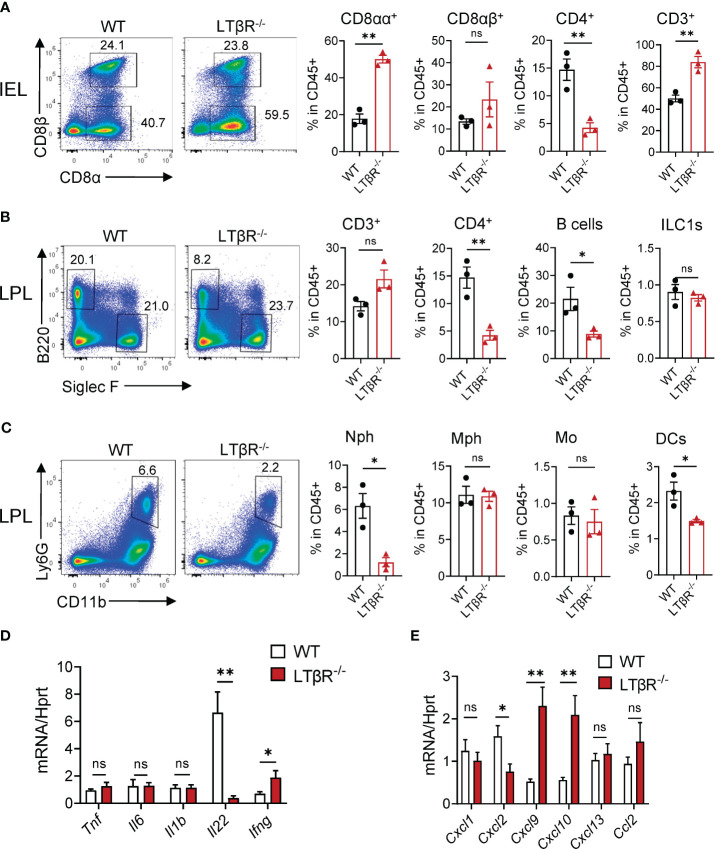
LTβR signaling controls accumulation of B cells, neutrophils and CD4^+^ T cells in the small intestine early after MTX treatment. WT and LTβR^-/-^ mice were treated as in **Figure 1A**. Small intestines were collected on day 2 for analysis. **(A)** Representative flow cytometry plots and frequency of T cell populations in SI IEL. Frequency is calculated in live CD45^+^ gate. **(B, C)** Representative flow cytometry plots and frequency of cell populations in LP. B cells (CD45^+^B220^+^); CD4^+^ T cells; CD3^+^ T cells; ILC1s (CD45^+^Ly6G^-^B220^-^SiglecF^-^TCRβ^-^CD64^-^NK1.1^+^); Neutrophils (Nph, Ly6G^+^ CD11b^+^); Macrophages (Mph, CD11c^-^Ly6G^-^SiglecF^-^CD11b^+^MHCII^+^CD64^+^); Monocytes (Mo, CD45^+^Ly6G^-^B220^-^SiglecF^-^TCRβ^-^CD64^+^MHCII^-^CD11b^+^CCR2^+^); Dendritic cells (DCs, CD45^+^Ly6G^-^B220^-^SiglecF^-^TCRβ^-^CD64^-^MHCII^+^CD11c^+^). **(D)** Cytokine and **(E)** chemokine expression in the ileum on day 2 measured by real-time PCR. Data is representative from one of two independent experiments with similar results (n=3–6 per group). Data shown as mean ± SEM. ns, not significant, *p<0.05, **p<0.01. Statistics were determined using t test **(A, B)** or ANOVA with Sidak’s multiple comparison test **(D, E)**. Gating strategy is shown in **Supplementary Figure S2**.

To define the impact of LTβR on the recruitment of immune cells after MTX treatment, we next analyzed immune cells in the SI of LTβR^-/-^ mice at day 2 and compared them to control WT mice. We found an increased frequency of T cells and CD8αα^+^ T cells in the SI IEL of LTβR^-/-^ mice (**Figure 2A**). We did not observe a difference in total T cell frequency in the SI LP isolated from LTβR^-/-^ mice; however, the frequency of CD4^+^ T cells, B cells, DCs, and neutrophils was reduced (**Figures 2B, C**). Correspondingly, mRNA expression of the neutrophil-recruiting chemokine CXCL2 was reduced in the ileum of LTβR^-/-^ mice on day 2 after MTX treatment (**Figures 2C, E**), in contrast to the increased levels of CXCL1, CXCL2 at day 5 post MTX treatment (**Figure 1I**). These results suggest that LTβR signaling controls early neutrophil recruitment after MTX-induced injury but is dispensable at later stages of the disease when inflammation is more pronounced. Similarly, we did not detect increased expression of proinflammatory cytokines TNF and IL-1β in the ileum of LTβR^-/-^ mice at day 2 (**Figure 2D**) in contrast to day 5 after MTX administration (**Figure 1H**); however, expression of IFNγ and IFNγ-dependent chemokines CXCL9 and CXCL10 was elevated (**Figures 2D, E**). Interestingly, LTβR^-/-^ mice failed to upregulate IL-22 expression early after MTX administration (**Figure 2D**), suggesting that LTβR signaling controls IL-22 production in this model of intestinal inflammation. Collectively, these results suggest that LTβR signaling inhibits inflammation during MTX-induced injury.

### LTβR ligand LIGHT is necessary for protection from MTX-induced intestinal damage

**Figure 3:**
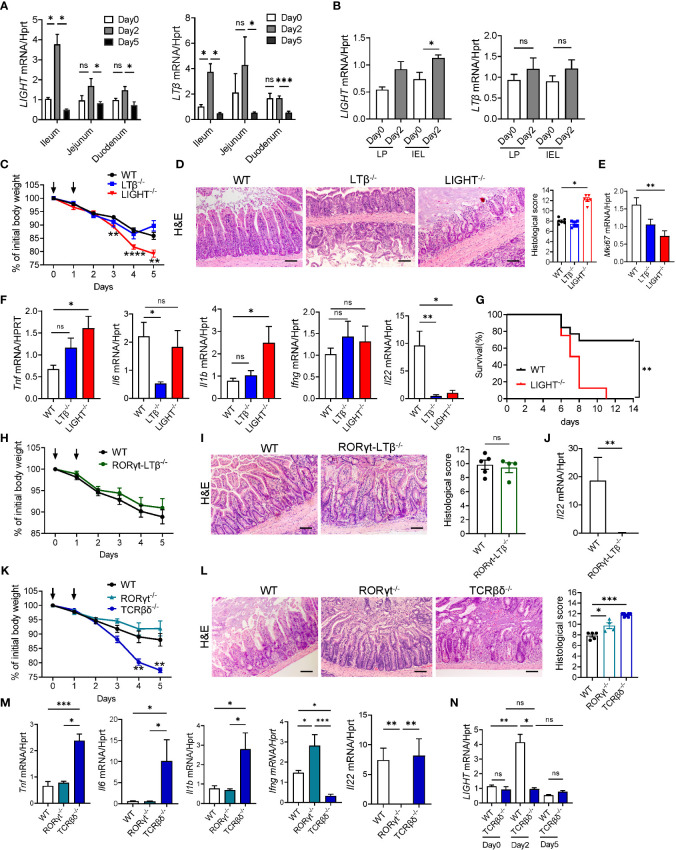
LIGHT and T cells protect against MTX-induced intestinal injury. **(A, B)** Kinetics of LIGHT and LTβ expression after MTX treatment in **(A)** ileum, jejunum and duodenum, and **(B)** LP and IEL from small intestine of WT mice. n=3–4 per group. **(C–F)** WT, LTβ^-/-^ and LIGHT^-/-^ mice were treated with MTX as in **Figure 1A**. **(C)** Body weight loss. Black arrows: days of MTX treatment. n=15–25 mice per group. **(D)** Representative H&E images (scale bars, 100μm) and histopathology scores; **(E)** Ki-67 and **(F)** cytokine expression in the ileum of WT, LTβ^-/-^ and LIGHT^-/-^ mice on day 5 after MTX treatment. n=6–8 mice per group. **(G)** Survival of LIGHT^-/-^ mice after MTX treatment. n=8–13 mice per group. **(H–J)** WT and RORγt-LTβ^-/-^ mice were treated with MTX as in **Figure 1A**. **(H)** Body weight loss; n=13–15 mice per group. **(I)** Representative H&E images (Scale bars, 100μm) and histopathology scores; and **(J)** IL-22 expression in the ileum on day 5 after MTX treatment. n=5 mice per group. **(K–M)** WT, RORγt^-/-^ and TCRβδ^-/-^ mice were treated with MTX as in **Figure 1A**. **(K)** Body weight loss; n=8–14 per group. **(L)** Representative H&E images (Scale bars, 100μm) and histopathology scores and **(M)** cytokine expression in the ileum on day 5 after MTX treatment. n= 7 mice per group. **(N)** LIGHT expression in the ileum of WT and TCRβδ^-/-^ mice at indicated time points after MTX treatment analyzed by real-time PCR. n=4–7 mice per group. H&E images and histopathology scores are representative from 3–4 independent experiments with similar results. Data shown as mean ± SEM. Statistics were determined using two-way ANOVA with Geisser-Greenhouse correction **(A, C, K)**, Mann-Whitney test **(B, J)**, Kruskal-Wallis test **(D–F, L, M)** or Brown-Forsythe and Welch ANOVA tests **(N)**. ns, not significant, *p<0.05, **p<0.01, ***p<0.001, ****p<0.0001.

LTβR signaling can be activated by two ligands, membrane-bound lymphotoxin (LTα1β2) and LIGHT (TNFSF14), both known regulators of intestinal inflammation (38, 40, 47, 64, 69). To test whether MTX treatment regulates expression of LTβR ligands, we analyzed expression of LIGHT and LTβ in the ileum, jejunum, and duodenum of WT mice during MTX treatment. Expression of both LIGHT and LTβ was significantly increased in the ileum on day 2 after MTX treatment and decreased at day 5 during resolution of MTX-induced injury (**Figure 3A**). Interestingly, expression of LIGHT was also increased in the LP and IEL fractions isolated from total small intestine on day 2 after MTX treatment, while we did not detect induction of LTβ expression (**Figure 3B**). Expression of LTα followed the same pattern as LTβ (**Supplementary Figure S3F, G**). To assess which LTβR ligand is essential for protection from MTX-induced injury, we treated WT, LTβ^-/-^ and LIGHT^-/-^ mice with MTX. While body weight loss in LTβ^-/-^ mice followed the same pattern as in WT mice, LIGHT^-/-^ mice lost significantly more body weight (**Figure 3C**), and all succumbed to the injury induced by MTX (**Figure 3G**). Consistently, histological analysis showed increased histopathology scores in the ileum of LIGHT^-/-^ mice, but not in LTβ^-/-^ mice, compared to WT controls (**Figure 3D**). Crypt regenerative capacity measured by expression of Ki-67 was markedly reduced in LIGHT^-/-^ mice (**Figure 3E**). To further examine the role of LTβR ligands in MTX- induced inflammation, we next measured the expression of proinflammatory cytokines in the ileum of MTX-treated mice on day 5. Expression of TNF and IL-1β was increased in the ileum of LIGHT^-/-^ but not LTβ^-/-^ mice, while IFNγ levels were not changed (**Figure 3F**). IL-6 expression was not changed in LIGHT^-/-^ mice but reduced in LTβ^-/-^ mice (**Figure 3F**). We also found that production of IL-22 was reduced in the ileum of both LTβ^-/-^ mice and LIGHT^-/-^ mice (**Figure 3F**). Collectively, these data suggest that whereas both LIGHT and lymphotoxin are upregulated in the small intestine during MTX-induced injury and both LTβR ligands contribute to IL-22 production, LIGHT, but not LT reduces inflammation and promotes intestinal healing during MTX-induced injury.

Previous studies revealed that LT produced by RORγt^+^ ILC in the intestine is critical for control of IL-22 production and protection of mice against *Citrobacter rodentium* infection (43, 47). Moreover, depletion of ILCs in Rag1^-/-^ mice resulted in reduced LTβ and IL-22 production in the ileum and diminished crypt proliferation during MTX treatment (30). To test whether LT produced by RORγt^+^ cells is essential for protection against MTX-induced damage, we utilized mice with specific inactivation of LTβ in RORγt-expressing cells (RORγt-LTβ^-/-^ mice) (47). Surprisingly, we did not find difference in body weight loss or histopathology score between RORγt-LTβ^-/-^ mice and littermate control LTβ floxed mice (**Figures 3H, I**) despite reduced expression of IL-22 in the ileum of RORγt-LTβ^-/-^ mice (**Figure 3J**). Thus, these results suggest that although LTβ produced by RORγt^+^ cells is required for IL-22 production in the gut, it is dispensable for control of intestinal damage during MTX-induced disease.

### T cell deficiency aggravates intestinal damage after MTX treatment

Recent studies implicated the role of RORγt^+^ ILCs in the maintenance of ISCs and intestinal repair following MTX-induced intestinal damage (30, 31). Our data demonstrated that CD3^+^ T cells are increased in the IEL after MTX treatment (**Supplementary Figure S3A**). To define the relative contribution of T cells and ILC3s in MTX-induced pathology we treated mice which lack ILC3s (RORγt^-/-^ mice) or T cells (TCRβδ^-/-^ mice) with MTX. RORγt^-/-^ mice displayed 5–10% of body weight loss similar to WT control mice, however histological analysis of the ileum demonstrated increased crypt loss and crypt flattening (**Figures 3K, L**). Unexpectedly, T cell-deficient mice lost more than 20% of body weight and had to be euthanized by day 5 of MTX treatment (**Figure 3K**). Histological analysis showed severe loss of crypts, increased inflammation, and bleeding (**Figure 3L**). Expression of proinflammatory cytokines TNF, IL-6 and IL-1β was increased in the ileum of TCRβδ^-/-^ mice but not RORγt^-/-^ mice (**Figure 3M**). These results suggest that T cells, but not ILC3s are critical for protection against MTX induced injury. Interestingly, IFNγ expression was very low in the ileum of TCRβδ^-/-^ mice, indicating that T cells are the main producers of IFNγ in the ileum after MTX treatment. We did not find a defect in IL-22 expression in the ileum of TCRβδ^-/-^ mice, however IL-22 transcript was almost undetectable in the ileum of RORγt^-/-^ mice (**Figure 3M**), implying that RORγt^+^ ILCs but not T cells are the main source of IL-22 production after MTX-induced injury.

Since LIGHT is mainly produced by activated T cells (37, 40) and LIGHT^-/-^ mice displayed increased intestinal pathology post MTX treatment (**Figures 3C–G**), we next analyzed kinetics of LIGHT expression in the ileum of TCRβδ^-/-^ mice during MTX treatment. While we did not find difference in LIGHT levels between WT and TCRβδ^-/-^ mice at steady-state, T cell-deficient mice failed to upregulate LIGHT in the ileum at day 2 post MTX treatment (**Figure 3N**). Thus, these data suggest that T cells are critical for protection from chemotherapy-induced intestinal injury and can serve as the primary source of LIGHT early after MTX-induced damage.

### LTβR and IL-22 jointly protect from MTX-induced intestinal damage

**Figure 4:**
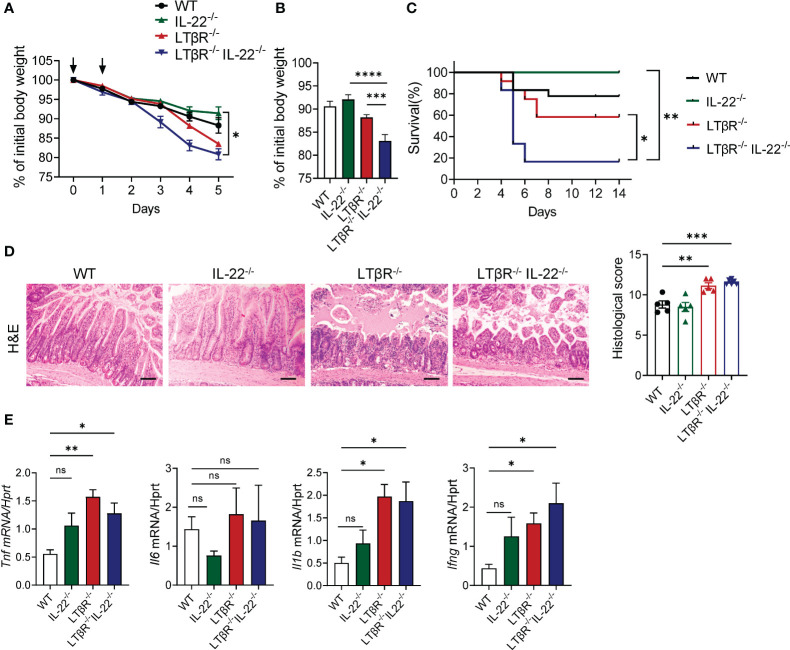
LTβR signaling cooperates with IL-22 for mucosal protection. LTβR^-/-^ mice were intercrossed with IL-22^-/-^ mice. Mice were treated with MTX as in **Figure 1A**. **(A)** Kinetics of body weight loss. n=8–18 mice per group. **(B)** Body weight loss at day 4 after MTX treatment. **(C)** Survival, n=6–18 mice per group. **(D)** Representative H&E images (scale bars, 100μm) and histopathology scores. **(E)** Expression of proinflammatory cytokines in the ileum on day 5. n=6–8 mice per group. Data was combined from 4–7 experiments with similar results. Statistics were determined using two-way ANOVA with Geisser-Greenhouse correction **(A)**, unpaired t test **(B)**, log-rank (Mantel-Cox) test **(C)**, ordinary one-way ANOVA **(D)**, Kruskal-Wallis test **(E)**. ns, not significant, *p<0.05, **p<0.01, ***p<0.001, ****p<0.0001.

IL-22 blockade during MTX-induced intestinal damage led to a significant loss of Lgr5^+^ stem cells, specifically in the duodenum (30), although crypt proliferation and crypt pathology in the small intestine of IL-22^-/-^ mice after MTX treatment was indistinguishable from WT controls (31). We found that IL-22 expression is induced in the ileum on day 2 after MTX treatment (**Supplementary Figure S3D**) and that IL-22 is downregulated in LTβR^-/-^ mice (**Figures 1K**, **2D**), suggesting that LTβR signaling regulates production of IL-22 during MTX-induced injury. To determine whether LTβR plays a protective role independently of IL-22, we intercrossed LTβR^-/-^ mice with IL-22^-/-^ mice and compared intestinal pathology in IL-22^-/-^ and LTβR^-/-^ mice with double deficient LTβR^-/-^IL-22^-/-^ mice after MTX administration. We did not find difference in body weight loss, survival, or intestinal pathology between IL-22^-/-^ and littermate heterozygous control WT mice (**Figures 4A–D**), consistent with previous studies (31). However, LTβR^-/-^IL-22^-/-^ mice displayed increased body weight loss, intestinal pathology and exacerbated mortality compared to IL-22^-/-^ mice (**Figures 4A–D**) suggesting that loss of LTβR exacerbates MTX-induced intestinal pathology in IL-22^-/-^ mice. Interestingly, body weight loss and mortality were exacerbated in LTβR^-/-^IL-22^-/-^ double deficient mice compared to LTβR^-/-^ mice (**Figures 4B, C**), suggesting that complete loss of IL-22 exacerbates MTX-induced pathology in LTβR^-/-^ mice. Consistently, LTβR^-/-^IL-22^-/-^ mice displayed increased levels of proinflammatory cytokines TNF, IL-1β, and IFNγ in the ileum compared to IL-22^-/-^ and WT control mice (**Figure 4E**). These results imply that LTβR and IL-22 jointly protect from MTX-induced intestinal damage and that LTβR may control both IL-22 dependent and IL-22 independent pathways for mucosal protection.

### LTβR signaling in epithelial cells is essential for mucosal repair following MTX-induced damage

**Figure 5:**
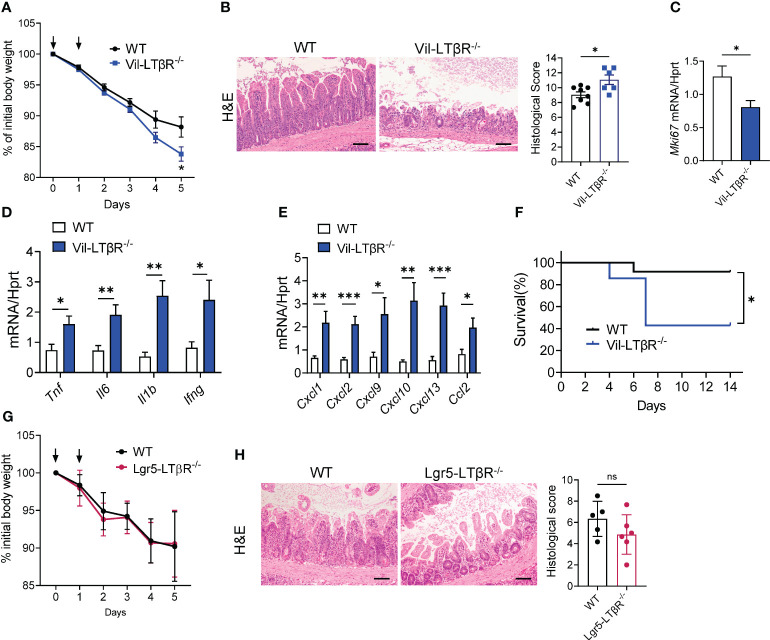
LTβR signaling in intestinal epithelial cells is required for protection against MTX-induced intestinal damage. **(A–F)** WT and Vil-LTβR^-/-^ mice were treated with MTX, as in **Figure 1A**. **(A)** Body weight loss; n=19–22 mice per group. **(B)** Representative H&E images (scale bars, 100μm) and histopathology scores; Expression of **(C)** Ki-67, **(D)** cytokines and **(E)** chemokines in the ileum on day 5 after MTX treatment. n= 6–8 mice per group. **(F)** Survival; n=7–12 mice per group. **(G, H)** LTβR expression by Lgr5^+^ cells is dispensable for protection. WT and Lgr5-LTβR^-/-^ mice were treated with MTX, as in **Figure 1A**, Lgr5-Cre expression was induced by tamoxifen administration. Mice were euthanized on day 5 for analysis. **(G)** Body weight change, n=11–14 mice per group **(H)** representative H&E images (scale bars, 100μm) and histopathology scores. Data combined from 2–5 independent experiments with similar results. Data shown as mean ± SEM. Statistics were determined using multiple unpaired t test **(A)**, Mann-Whitney test **(B)**, unpaired t test **(C–E, H)** or Log-rank (Mantel-Cox) test **(F)**. ns, not significant; *p<0.05, **p<0.01, ***p<0.001.

Next, we sought to determine which LTβR-expressing cells are important for protection against MTX-induced epithelial injury. Since previous studies highlighted the role of LTβR signaling in intestinal epithelial cells for protection against epithelial injury caused by bacterial infection or by chemical agent (43, 45, 59), we tested whether LTβR signaling in epithelial cells is essential for mucosal repair during MTX-induced damage. Therefore, we generated mice with specific inactivation of LTβR in intestinal epithelial cells (Vil-LTβR^-/-^ mice) by crossing LTβR floxed mice (45) with Villin-Cre (51). Vil-LTβR^-/-^ mice demonstrated an accelerated body weight loss and increased mortality after MTX treatment, compared to littermate Cre-negative control mice (**Figures 5A, F**). Histological analysis and analysis of Ki-67 expression revealed increased tissue damage and reduced epithelial cell proliferation in the ileum of Vil-LTβR^-/-^ mice compared to control mice (**Figures 5B, C**). Expression of proinflammatory cytokines TNF, IL-6, IL-1β and IFNγ was increased in the ileum of Vil-LTβR^-/-^ mice on Day 5 (**Figure 5D**). Additionally, we found increased expression of CXCL1, CXCL2, CXCL9, CXCL10, CXCL13 and CCL2 chemokines in the ileum of Vil-LTβR^-/-^ mice (**Figure 5E**). These results demonstrate that LTβR signaling in intestinal epithelial cells is essential for protection against MTX-induced injury.

Regeneration of intestinal epithelium after damage depends on continuous differentiation of epithelial cells from ISCs (70). Lgr5^+^ ISCs have the ability to give rise to all intestinal epithelial cells (71). The maintenance of ISCs after intestinal damage is dependent on IL-22 production by ILC3s (26, 30). Since LTβR signaling controls IL-22 production by ILC3s in several models of intestinal inflammation (45, 47), we sought to determine whether LTβR signaling in Lgr5^+^ ISCs directly contributes to epithelium regeneration after MTX-induced injury. Therefore, we generated Lgr5-LTβR^-/-^ mice by crossing LTβR floxed mice (45) with Lgr5-EGFP-IRES-CreERT2 mice (54), and treated them with MTX. However, Lgr5-LTβR^-/-^ mice did not show increased weight loss or aggravated intestinal pathology, compared to littermate Cre^-^ control mice (**Figures 5G, H**). Moreover, analysis of publicly available single-cell RNA-sequence survey of the small intestine epithelium in naïve WT mice (72) revealed that while LTβR was highly expressed in goblet cells and enterocytes, LTβR expression was low-to moderate in Lgr5^hi^ ISC, TA.G2 or Paneth cells (**Supplementary Figure S4A**). Furthermore, to test whether global LTβR deficiency affects maintenance and/or proliferation of ISCs after mucosal damage, we intercrossed LTβR^-/-^ mice with Lgr5-EGFP-IRES-CreERT2 reporter mice and analyzed epithelial cell populations in the ileum on day 5 after MTX treatment. We did not find significant difference in the ratio of Lgr5^+^ ISCs, Paneth cells, tuft cells, epithelial cells, goblet cells between control and LTβR^-/-^ mice (**Supplementary Figures S4B–G**). Collectively, these data suggest that LTβR signaling is dispensable for ISC maintenance and proliferation after MTX-induced injury.

Previous studies have implicated the role of LTβR signaling in CD11c^+^ DCs for IL-22 production and mucosal protection against intestinal bacterial infection (47). In addition, LTβR expression in neutrophils contributes to mucosal repair in DSS-induced colitis (46). To define whether expression of LTβR in DCs and macrophages/monocytes contributes to protection from MTX-induced injury, we treated mice with CD11c^+^ DC-specific deficiency of LTβR (CD11c-LTβR^-/-^mice) (45) and macrophage/neutrophil-specific LTβR deficiency (LysM-LTβR^-/-^ mice) (43), as well as mice with combined deficiency (CD11c, LysM-LTβR^-/-^) with MTX, and then analyzed body weight loss and pathology on day 5 after MTX administration. We did not find difference in body weight loss or intestinal pathology in any of these strains, compared to Cre^-^ littermate controls (**Supplementary Figures S5A, B**). Interestingly, IL-22 expression was decreased in the ileum of CD11c-LTβR^-/-^ mice (**Supplementary Figure S5C**). This decrease suggests that while LTβR signaling in CD11c^+^ cells is not critical for control of intestinal injury after MTX treatment, it may contribute to the IL-22-dependent maintenance of ISCs. Collectively, our data suggest that LTβR signaling in epithelial cells, but not immune cells is essential for protection from MTX-induced intestinal damage.

### Non-canonical NF-κB signaling in intestinal epithelial cells protects from MTX-induced intestinal damage

**Figure 6:**
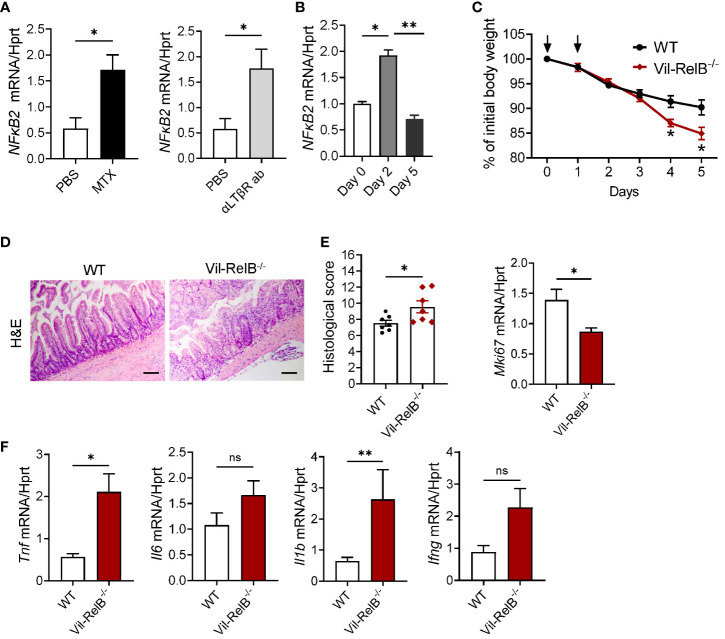
Non-canonical NF-κB signaling in intestinal epithelial cells protects from MTX-induced intestinal damage. **(A)** CMT-93 epithelial cells were treated with MTX (5 μmol/L) or agonistic αLTβR antibody (ACH6, 0.5μg/ml) for 24 hours. Nfκb2 expression was measured by real-time PCR. **(B)** Nfκb2 expression in the ileum of WT mice treated with MTX was measured by real-time PCR. n= 4–7 mice per group. **(C–F)** WT and Vil-RelB^-/-^ mice were treated with MTX as on **Figure 1A**. **(C)** Body weight loss; n=14–30 per group. **(D)** representative H&E images (scale bars, 100μm) and histopathology scores; Expression of **(E)** Ki-67, and **(F)** proinflammatory cytokines in the ileum on day 5 after treatment. n= 5 mice per group. Data is combined from 3–4 independent experiments with similar results. Data shown as mean ± SEM. Statistics were determined using unpaired t test **(A, D)**, Mann-Whitney test **(A, B)**, two-way ANOVA with Geisser-Greenhouse correction **(C)**, or Kruskal-Wallis test **(E, F)**. ns, not significant; *p<0.05; **p<0.01.

**Figure 7:**
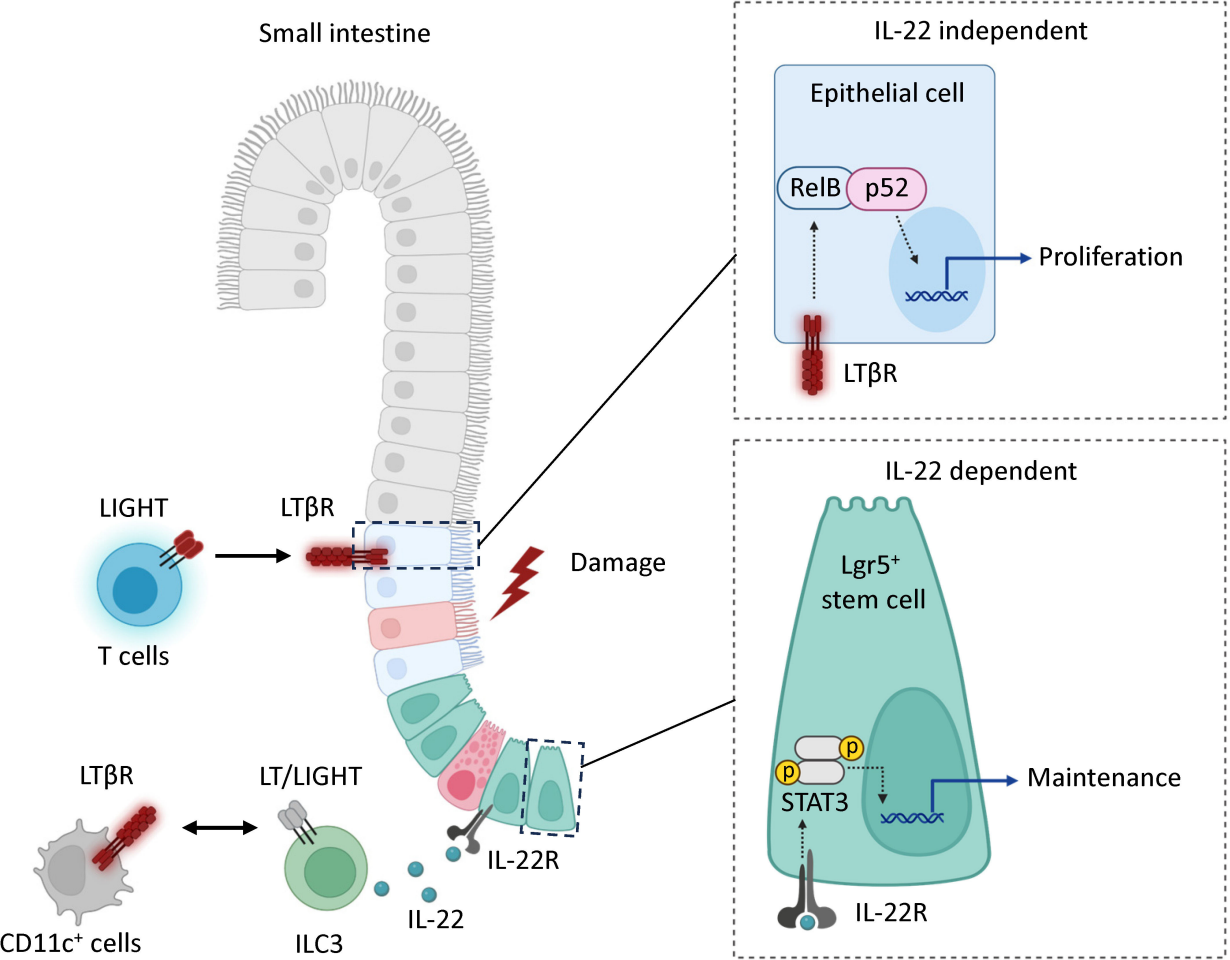
Model. LTβR signaling promotes mucosal healing following MTX-induced injury by controlling IL-22 dependent and IL-22 independent pathways. Mucosal damage promotes expression of LIGHT and LTβ in the intestine. In the IL-22 independent pathway, the interaction of LIGHT expressing T cells with LTβR in intestinal epithelial cells activates non-canonical RelB/p52 NF-kB signaling to promote proliferation of epithelial cells after injury. In the IL-22 dependent pathway, interaction of LIGHT/LT expressing ILC3s with CD11c^+^ LTβR-expressing cells promotes secretion of IL-22 which interacts with IL-22R to support the maintenance of Lgr5^+^ intestinal stem cells. LTβR expression in Lgr5^+^ stem cells is dispensable for protection.

As our experiments with LTβR^-/-^IL-22^-/-^ mice (**Figure 4**) implied that LTβR-dependent IL-22- independent signaling could contribute to protection from MTX-induced damage, we next tested whether LTβR-dependent regulation of the NF-κB pathway is important for mucosal healing. LTβR signaling can activate both canonical and non-canonical NF-κB signaling pathways to produce various proinflammatory cytokines and chemokines in response to the inflammatory stimuli (39, 42, 73). NF-κB signaling in intestinal epithelial cells can contribute to protection from intestinal inflammation in several animal models of disease (74). Recent studies demonstrated the important role of non-canonical NF-κB signaling in intestinal epithelial cells for protection from gut bacterial infections and intestinal inflammation (59, 75). We found that treatment of CMT-93 intestinal epithelial cells *in vitro* with MTX or with an agonistic αLTβR antibody induced expression of NF-κB2 (**Figure 6A**). Moreover, NF-κB2 was upregulated in the ileum after MTX treatment *in vivo* (**Figure 6B**). To test whether non-canonical NF-κB signaling in intestinal epithelial cells protects from intestinal inflammation caused by MTX treatment, we generated mice with specific inactivation of RelB in intestinal epithelial cells (Vil-RelB^-/-^ mice) by crossing RelB floxed mice (55) with Villin-Cre mice (51). Vil-RelB^-/-^ mice treated with MTX demonstrated aggravated weight loss and increased intestinal pathology, compared to littermate Cre^-^ control mice (**Figures 6C, D**). Whereas proliferation of intestinal epithelial cells in these mice was decreased (**Figure 6E**), expression of proinflammatory cytokines TNF and IL-1β was elevated (**Figures 6F**). In contrast to Vil-RelB^-/-^, mice with inactivation of RelB in CD11c^+^ DCs (CD11c-RelB^-/-^) did not display an increased body weight loss post MTX treatment (**Supplementary Figure S5D**), consistent with results in CD11c-LTβR^-/-^ mice (**Supplementary Figures S5A, B**). Together, these results suggest that LTβR on intestinal epithelial cells activates non-canonical NF-κB signaling to promote recovery after MTX-induced injury (**Figure 7**).

## Discussion

Accumulating evidence suggests that immune mechanisms may either exacerbate or ameliorate intestinal damage caused by chemotherapeutic drugs. Recent studies implicated the role of IL-22 and ILC3s in mucosal repair following MTX-induced intestinal damage (30, 31), however the role of other immune components and cytokines remains less defined. In this study we revealed the critical role of LTβR in protection from chemotherapy-induced intestinal damage. As previous studies demonstrated the role of LTβR in regulation of IL-22 production by ILC3s (45, 47), we hypothesized that LTβR-dependent regulation of ILC3s and IL-22 mediates protection against chemotherapy-induced intestinal damage. However, our results suggest that although LT expression in ILC3s is critical for control of IL-22 production, it is dispensable for protection from MTX-induced injury. Instead, another LTβR ligand, LIGHT, produced by T cells was critical for protection. Moreover, LTβR and IL-22 pathways jointly participate in mucosal protection. Furthermore, we demonstrate that LTβR-dependent non-canonical NF-kB signaling in intestinal epithelial cells is required for mucosal repair.

Although the role of LTβR signaling in the development and maintenance of lymphoid tissues and inflammatory diseases is well established (41, 76–78), accumulating evidence suggests that LTβR regulates intestinal inflammation (43, 45–47, 64, 79, 80). However, the role of LTβR in chemotherapy-induced epithelial injury has not been investigated. Our data demonstrate that LTβR-deficient mice display increased body weight loss, severe pathology, reduced epithelial cell proliferation and increased mortality post MTX administration. This phenotype was associated with increased expression of proinflammatory cytokines TNF, IL-1β, IFNγ and chemokines CXCL1, CXC2, CXCL9, CXCL10, and CCL2 in the small intestine at day 5 post MTX administration, whereas IL-22 and IL-22 dependent expression of antibacterial proteins were reduced. These results are consistent with previous studies supporting the role of LTβR in regulation of colonic IL-22 production and protection against *C. rodentium* infection (44, 47). As increased expression of proinflammatory cytokines at day 5 can be a result of impaired epithelial cell proliferation, we next analyzed immune cell populations and cytokines at day 2 post MTX administration, during the disease induction phase. Our results show that expression of CXCL2 and IL-22 was reduced in the ileum of LTβR^-/-^ mice, whereas IFNγ, CXCL9, CXCL10 were increased at day 2 post MTX treatment. This is consistent with LTβR function in controlling neutrophil recruiting chemokines in response to mucosal bacterial pathogen *C. rodentium* (43). Flow cytometry revealed an increased frequency of CD8αα^+^ IELs whereas proportion of CD4^+^ T cells was reduced in the IEL and LP of LTβR^-/-^ mice. CD8αα^+^ IELs are known to play regulatory role in intestinal inflammation (81, 82). How LTβR signaling controls CD8αα^+^ IELs recruitment and the role of these cells in chemotherapy-induced epithelial damage remains to be determined.

Both LTβR ligands LT and LIGHT have been implicated in the regulation of inflammatory responses in the gut (43, 69, 79). Surprisingly, LIGHT but not LTβ, was essential for protection from MTX-induced intestinal damage, as LIGHT^-/-^ mice displayed increased intestinal pathology post MTX treatment whereas LTβ^-/-^ mice did not exhibit an exacerbated pathology. Furthermore, inactivation of LTβ in ILC3s did not result in increased intestinal pathology, despite reduced IL-22 levels in the ileum of RORγt-LTβ^-/-^ mice. These results highlight distinct roles of LIGHT and LT in different models of intestinal damage. Thus, LTβ expressed by RORγt^+^ ILC3s is critical for protections against *C. rodentium*, while LIGHT is dispensable in this model (47). In contrast, LIGHT, rather than LTβ, was critical for protection against DSS-induced intestinal damage (69, 79). Interestingly, LIGHT^-/-^ mice displayed reduced levels of IL-22 in the ileum post MTX treatment, suggesting that LIGHT signaling can also control IL-22 production in this model of intestinal damage. In contrast, in the *C. rodentium* colitis model, LTβ, but not LIGHT, was critical for IL-22 production (47). The distinct role of LIGHT and LTβ in these models of intestinal damage could be attributed to different LIGHT and LTβ producing cell types. Our data revealed that T cells are the major contributors to mucosal protection against MTX induced damage, because TCRβδ^-/-^ mice displayed an exacerbated intestinal pathology compared to RORγt^-/-^ mice. As LIGHT expression was rapidly increased in the intestine at day 2 post MTX treatment, but was ablated in TCRβδ^-/-^ mice, this data suggest that LIGHT provided by T cells contribute to mucosal protection. It is also possible that LIGHT expression by other immune or stromal cells contribute to protection. Our results are in line with a previous study suggesting the role of LIGHT in regulation of intestinal stem cell gene signatures (83). The kinetics and level of LIGHT expression may explain protective versus pathogenic LIGHT-mediated responses in the gut. Consistent with this hypothesis, we detected only a transient induction of LIGHT expression in the MTX-induced injury model. In contrast, sustained overexpression of LIGHT on T cells can break down the immunosuppressive state mediated by Tregs and induce T cell- mediated intestinal inflammation (84, 85).

Previous studies demonstrated the critical role of IL-22 in promoting ISC proliferation after injury (19, 26, 29, 30). However, a recent study demonstrated that IL-22 deficient mice do not display increased intestinal pathology after MTX treatment, implicating IL-22 independent pathways, such as Hippo-Yap, in promoting intestinal epithelial cell proliferation after injury (31). Consistently, our study also did not detect an increased intestinal pathology in IL-22^-/-^ mice post MTX treatment. Although IL-22 expression was impaired in the ileum of RORγt-LTβ^-/-^ mice, these mice did not exhibit increased intestinal pathology. However, we revealed that genetic inactivation of IL-22 further exacerbated MTX-induced intestinal pathology in LTβR^-/-^ mice. These results suggest that LTβR and IL-22 jointly promote mucosal repair after MTX-induced intestinal damage. Interestingly, LTβR stimulation may suppress YAP/TAZ activity in fibroblastic reticular cells in lymph nodes (86). However, the connection between LTβR and Yap signaling in intestinal epithelial cells remains to be determined.

LTβR is expressed on a variety of epithelial, stromal, and myeloid cells in the gut, thereby participating in regulation of mucosal immune homeostasis (43, 45, 46, 59, 64, 87). Therefore, we wanted to determine which LTβR expressing cells are critical for protection against MTX-induced damage. Our results suggest that LTβR expression in intestinal epithelial cells is essential for protection, whereas LTβR expression on macrophages and dendritic cells is dispensable. The protective role of LTβR on intestinal epithelial cells was previously demonstrated in *C. rodentium* infection and DSS-induced colitis models (43, 45). However, genetic inactivation of LTβR in ISCs did not exacerbate intestinal disease, consistent with low expression of LTβR on Lgr5^+^ stem cells (72). These results suggest that although LTβR on intestinal epithelial cells is critical for mucosal repair after MTX-induced damage, LTβR signaling in ISCs is dispensable for protection. The role of specific subsets of LTβR-expressing intestinal epithelial cells in mucosal repair after MTX-induced damage will be further defined in future studies.

LTβR stimulation leads to non-canonical NF-kB signaling, which involves NF-κB-inducing kinase (NIK) and IKKα, processing of p100 precursor and nuclear translocation of the non-canonical NF-κB complex p52/RelB (39, 42, 88). Additionally, LTβR stimulation can lead to activation of the canonical NF-κB pathway operating via NFκB1 (p50/RelA) transcription, which usually occurs within minutes and does not require novel gene expression, in contrast to the non-canonical pathway (73, 88). Non-canonical NF-kB signaling is thought to play a central role in induction of proinflammatory cytokines TNF, IL-6, IL-18, IL-1β early during chemotherapy-induced intestinal injury, thereby promoting inflammation (2, 3). In contrast, non-canonical NF-κB signaling in intestinal epithelial cells is important for protection from gut bacterial infections and intestinal inflammation (59, 74, 75). Our results are consistent with these studies and identify a previously unrecognized role for epithelial cell-intrinsic RelB expression in regulating mucosal repair after chemotherapy-induced intestinal damage.

Based on our results, we propose a model for a LTβR-dependent mechanism for mucosal healing after MTX-induced intestinal damage (**Figure 7**). MTX injury results in early upregulation of chemokines and increased recruitment of T cells to the epithelial layer. LIGHT, presumably produced by T cells interacts with LTβR on intestinal epithelial cells to activate non-canonical RelB signaling thereby promoting proliferation of epithelial cells after injury. Interactions between LIGHT/LT expressing RORγt^+^ ILC3s and LTβR expressing CD11c^+^ cells can also contribute to IL-22-dependent maintenance of ISCs after injury. Our data suggest that LTβR also promotes mucosal healing in 5-FU induced intestinal mucositis. The critical LTβR expressing cells and LTβR ligands in 5-FU induced intestinal injury remain to be determined.

Gaining insight into the immune regulation of mucosal healing post-cytotoxic drug exposure holds crucial implications for developing targeted therapeutic interventions. In summary, our study revealed a previously unrecognized role for the LTβR-RelB pathway in intestinal epithelial cells which promotes mucosal repair after chemotherapy-induced intestinal damage. These findings provide valuable insights into the immune mechanisms orchestrating mucosal healing after chemotherapy-induced intestinal injury, paving the way for potential therapeutic interventions.

## Data availability statement

Publicly available datasets were analyzed in this study. This data can be found here: https://portals.broadinstitute.org/single_cell/study/small-intestinal-epithelium.

## Ethics statement

The animal study was approved by The University of Texas Health Science Center at San Antonio Institutional Animal Care and Use Committee. The study was conducted in accordance with the local legislation and institutional requirements.

## Author contributions

AVT: Conceptualization, Formal analysis, Funding acquisition, Investigation, Methodology, Project administration, Resources, Supervision, Visualization, Writing – original draft, Writing – review & editing. QC: Data curation, Formal analysis, Investigation, Methodology, Writing – original draft, Writing – review & editing. AM: Conceptualization, Formal analysis, Investigation, Supervision, Writing – review & editing. AK: Data curation, Formal analysis, Investigation, Visualization, Writing – review & editing. YS: Investigation, Writing – original draft. JV: Investigation, Writing – original draft. AWT: Investigation, Writing – review & editing. SS: Data curation, Formal analysis, Investigation, Writing – review & editing. EK: Conceptualization, Data curation, Formal analysis, Investigation, Methodology, Project administration, Resources, Supervision, Validation, Writing – original draft, Writing – review & editing.

## Supplementary material

Supplementary Figure 1 LTβR signaling protects against 5-FU induced intestinal inflammation. WT and LTβR^-/-^ mice were treated with 5-Fluorouracil (5-FU, 50 mg/kg, i.p.) daily for 4 consecutive days, and analyzed at day 5. **(A)** Body weight change. Black arrows: days of 5-FU treatment. n=14–17 mice per group. **(B)** Disease score. **(C)** Representative photographs of colons and colon length. **(D)** Representative H&E images and histological scores. Scale bars, 100μm. **(E)** Cytokine expression in the colon. n= 7 mice per group. Data represents 1 of 3 independent experiments with similar results. Data shown as mean ± SEM. Statistics were determined using two-way ANOVA with Geisser-Greenhouse correction **(A)**, unpaired t test **(B–E)**. ns, not significant; * p<0.05, ** p<0.01, *** p<0.001, **** p<0.0001.

Supplementary Figure 2 Gating strategy of immune cell populations in SI. **(A)** Gating strategy of immune cell populations in IEL. Lin^+^(Lineage^+^): B220, Ly6G. Neutrophils were defined as CD45^+^Lin^+^MHCII-CD11b^+^; B cells were defined as CD45^+^Lin^+^CD11b-MHCII^+^; T cells were defined as CD45^+^CD3^+^ **(B)** Gating strategy of immune cell populations in LP. Neutrophils, CD45^+^Ly6G^+^CD11b^+^; B cells, CD45^+^Ly6G-B220^+^; Eosinophils, CD45^+^Ly6G^-^B220^-^CD11b^+^Siglec F^+^; T cells, CD45^+^Ly6G^-^B220^-^SiglecF^-^TCRβ^+^; ILC1s, CD45^+^Ly6G^-^B220^-^SiglecF^-^TCRβ^-^CD64^-^NK1.1^+^; Dendritic cells (DCs), CD45^+^Ly6G^-^B220^-^SiglecF^-^TCRβ^-^CD64^-^MHCII^+^CD11c^+^; Macrophages (Mph), CD45^+^Ly6G^-^B220^-^SiglecF^-^TCRβ^-^CD64^+^MHCII^+^CD11b^+^; Monocytes (Mo), CD45^+^Ly6G^-^B220^-^SiglecF^-^TCRβ^-^CD64^+^MHCII^-^CD11b^+^CCR2^+^.

Supplementary Figure 3 Analysis of immune cell populations and cytokines in WT SI after MTX treatment. **(A–G**) WT mice were treated with MTX as in **Figure 1A**. Mice were euthanized on day 2 and small intestines were collected for analysis. **(A)** Representative flow cytometry plots and frequency of T cell populations in SI IEL. Frequency is calculated in live CD45^+^ cells. **(B, C)** Representative flow cytometry plots and frequency of cell populations in LP. Tregs (B220^-^CD3^+^CD4^+^CD25^+^FoxP3^+^); ILC1s (CD45^+^Ly6G^-^B220^-^SiglecF^-^TCRβ^-^ CD64^-^NK1.1^+^); CD4^+^ T cells; CD3^+^ T cells; Macrophages (Mph, CD11c^-^Ly6G^-^SiglecF^-^CD11b^+^MHCII^+^CD64^+^); Neutrophils (Nph, Ly6G^+^ CD11b^+^); B cells (B220^+^); DCs (CD45^+^Ly6G^-^B220^-^SiglecF^-^TCRβ^-^CD64^-^MHCII^+^CD11c^+^). Expression of **(D)** cytokines and **(E)** chemokines in the ileum at day 0, 2 and 5 post MTX treatment. **(F, G)** LTα expression after MTX was measured by Real-Time PCR in WT **(F)** ileum, jejunum, and duodenum as well as **(G)** LP and IEL from small intestine. (**D–G)** Data are representative of two experiments (n=3–7 per group). Data shown as mean ± SEM. Statistics were determined using unpaired t test **(A–C, F, G)**, Mann-Whitney test **(D, E)**, Kruskal-Wallis test **(D–F)**. ns, not significant, * p<0.05, ** p<0.01, *** p<0.001, **** p<0.0001.

Supplementary Figure 4 Analysis of epithelial cell populations in the ileum of WT and LTβR^-/-^ mice. **(A)** Expression of *Ltbr* and *Lgr5* in various small intestine derived cell types was determined by single-cell RNA-seq. Data was obtained from the study conducted by Haber et al. (72), using the Broad Institute Single-Cell Portal for data analysis (https://portals.broadinstitute.org/single_cell/study/small-intestinal-epithelium). **(B)** WT and LTβR^-/-^ mice were crossed with Lgr5-GFP reporter mice. GFP expression was induced by tamoxifen administration and mice were treated with MTX as in **Figure 1A**. Mice were euthanized on day 5 and ileum epithelial cells analyzed by flow cytometry. Gating strategy. Tuft cells: EpCAM^+^CD45^+^; Lgr5^+^ cells: EpCAM^+^Lgr5^+^CD31^-^Ter119^-^CD45^-^; Paneth cells: EpCAM^+^c-Kit^+^CD31^-^Ter119^-^CD45^-^; Epithelial cells: EpCAM^+^CD31^-^Ter119^-^CD45^-^. **(C–F)** Representative flow plot and frequency of cell populations. **(G)** Goblet cells analysis by Alcian Blue staining in small intestine. Scale bars, 100μm. Data show 1 of 2 independent experiments with similar results (n=3–5 per group). Data shown as mean ± SEM. Statistics were determined using unpaired t test. ns, not significant.

Supplementary Figure 5 LTβR signaling in macrophages and DCs is not essential for the protection from MTX induced intestinal injury. **(A–D)**. WT, LysM-LTβR^-/-^, CD11c-LTβR^-/-^, CD11c, LysM-LTβR^-/-^ and CD11c-RelB^-/-^ mice were treated with MTX as in **Figure 1A**. **(A, D)** Body weight loss (n=9–14 mice per group) and **(B)** Representative H&E images (scale bars, 100μm) with histopathology scores. **(C)** IL-22 expression in the ileum on day 5 after MTX treatment. n= 5–8 mice per group. Data are combined from 3–5 independent experiments with similar results. Data shown as mean ± SEM. Statistics were determined using two-way ANOVA with Geisser-Greenhouse correction **(A, D)**, Kruskal-Wallis test **(B)**, unpaired t test **(C)**. ns, not significant, * p<0.05.
