# Supplementary figures and images for: LTβR-RelB signaling in intestinal epithelial cells protects from chemotherapy-induced mucosal damage
Source: Front Immunol. 2024 May 30;15:1388496. doi: 10.3389/fimmu.2024.1388496 (PMC11169669; doi:10.3389/fimmu.2024.1388496)

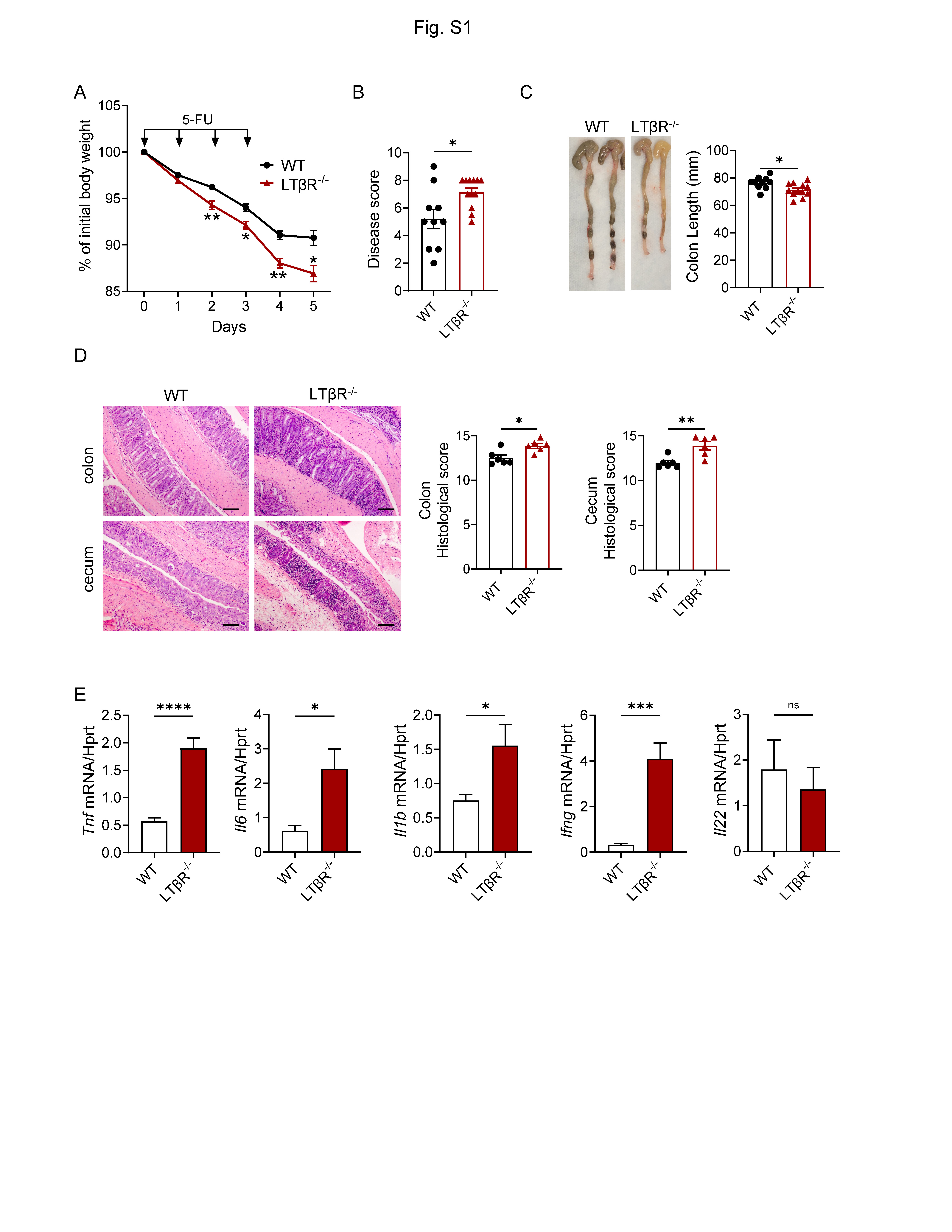

Supplement: Supplementary Figure 1 — LTβR signaling protects against 5-FU induced intestinal inflammation. WT and LTβR-/- mice were treated with 5-Fluorouracil (5-FU, 50 mg/kg, i.p.) daily for 4 consecutive days, and analyzed at day 5. (A) Body weight change. Black arrows: days of 5-FU treatment. n=14–17 mice per group. (B) Disease score. (C) Representative photographs of colons and colon length. (D) Representative H&E images and histological scores. Scale bars, 100μm. (E) Cytokine expression in the colon. n= 7 mice per group. Data represents 1 of 3 independent experiments with similar results. Data shown as mean ± SEM. Statistics were determined using two-way ANOVA with Geisser-Greenhouse correction (A), unpaired t test (B–E). ns, not significant; * p<0.05, ** p<0.01, *** p<0.001, **** p<0.0001. [file Image_1.jpeg]

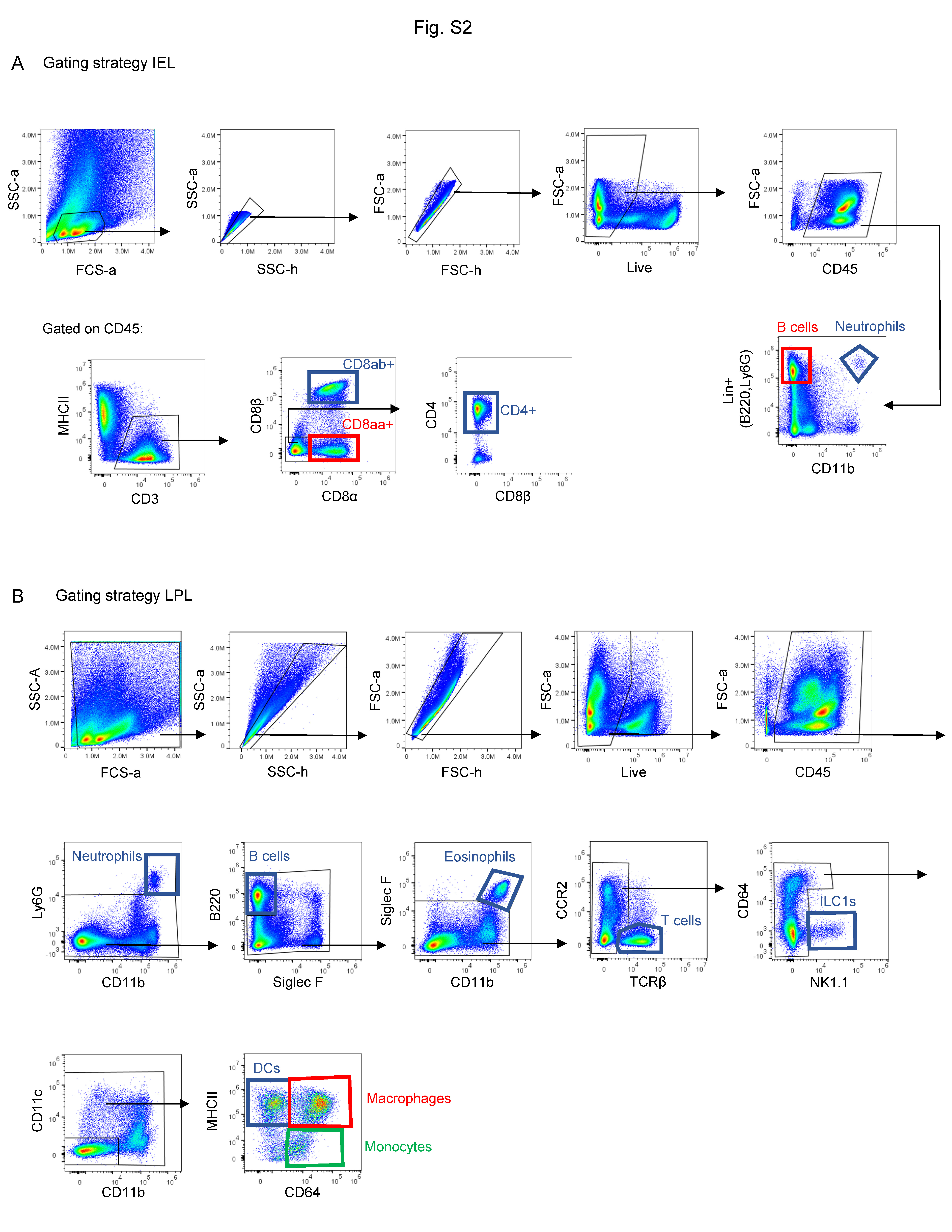

Supplement: Supplementary Figure 2 — Gating strategy of immune cell populations in SI. (A) Gating strategy of immune cell populations in IEL. Lin+(Lineage+): B220, Ly6G. Neutrophils were defined as CD45+Lin+MHCII-CD11b+; B cells were defined as CD45+Lin+CD11b-MHCII+; T cells were defined as CD45+CD3+ (B) Gating strategy of immune cell populations in LP. Neutrophils, CD45+Ly6G+CD11b+; B cells, CD45+Ly6G-B220+; Eosinophils, CD45+Ly6G-B220-CD11b+Siglec F+; T cells, CD45+Ly6G-B220-SiglecF-TCRβ+; ILC1s, CD45+Ly6G-B220-SiglecF-TCRβ-CD64-NK1.1+; Dendritic cells (DCs), CD45+Ly6G-B220-SiglecF-TCRβ-CD64-MHCII+CD11c+; Macrophages (Mph), CD45+Ly6G-B220-SiglecF-TCRβ-CD64+MHCII+CD11b+; Monocytes (Mo), CD45+Ly6G-B220-SiglecF-TCRβ-CD64+MHCII-CD11b+CCR2+. [file Image_2.jpeg]

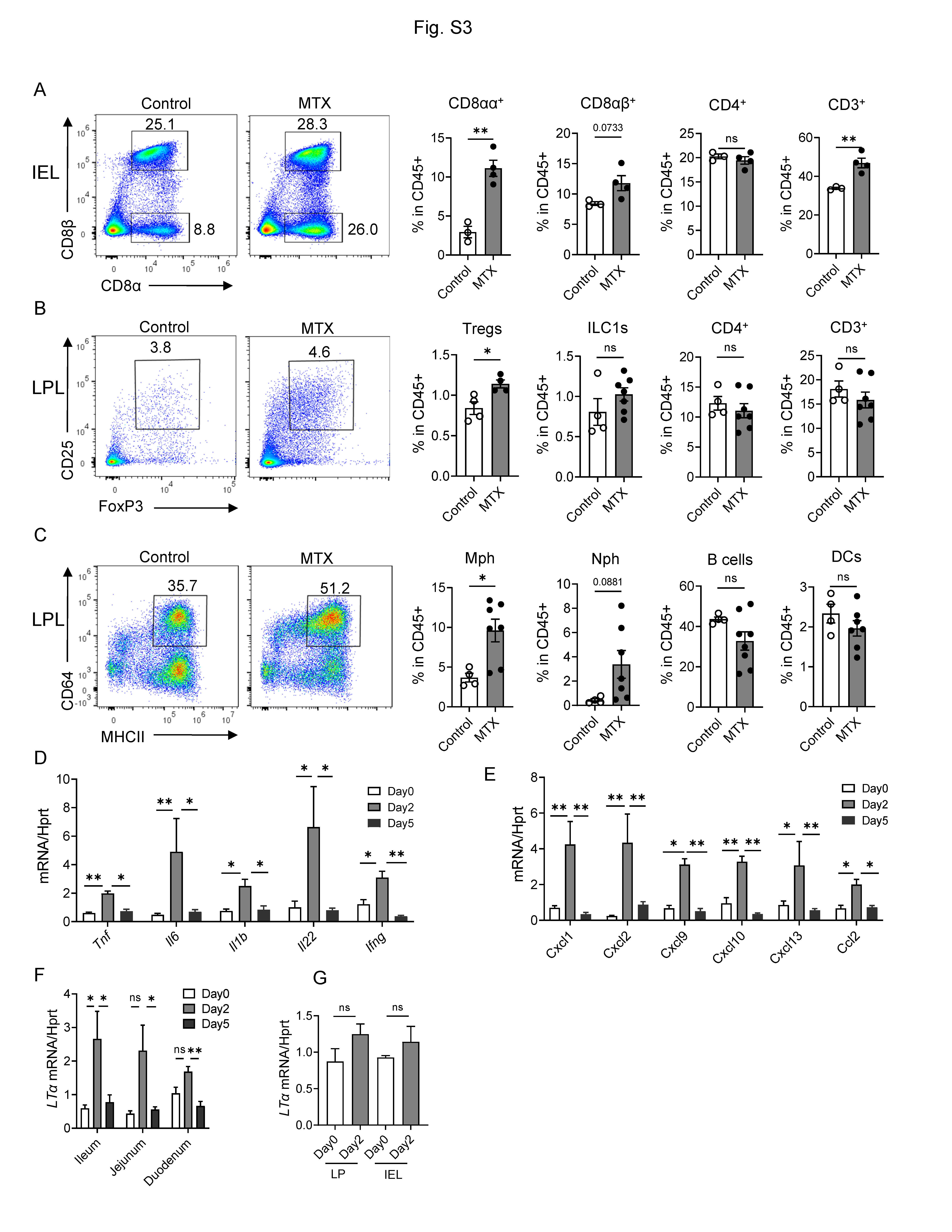

Supplement: Supplementary Figure 3 — Analysis of immune cell populations and cytokines in WT SI after MTX treatment. (A–G) WT mice were treated with MTX as in Figure 1A. Mice were euthanized on day 2 and small intestines were collected for analysis. (A) Representative flow cytometry plots and frequency of T cell populations in SI IEL. Frequency is calculated in live CD45+ cells. (B, C) Representative flow cytometry plots and frequency of cell populations in LP. Tregs (B220-CD3+CD4+CD25+FoxP3+); ILC1s (CD45+Ly6G-B220-SiglecF-TCRβ- CD64-NK1.1+); CD4+ T cells; CD3+ T cells; Macrophages (Mph, CD11c-Ly6G-SiglecF-CD11b+MHCII+CD64+); Neutrophils (Nph, Ly6G+ CD11b+); B cells (B220+); DCs (CD45+Ly6G-B220-SiglecF-TCRβ-CD64-MHCII+CD11c+). Expression of (D) cytokines and (E) chemokines in the ileum at day 0, 2 and 5 post MTX treatment. (F, G) LTα expression after MTX was measured by Real-Time PCR in WT (F) ileum, jejunum, and duodenum as well as (G) LP and IEL from small intestine. (D–G) Data are representative of two experiments (n=3–7 per group). Data shown as mean ± SEM. Statistics were determined using unpaired t test (A–C, F, G), Mann-Whitney test (D, E), Kruskal-Wallis test (D–F). ns, not significant, * p<0.05, ** p<0.01, *** p<0.001, **** p<0.0001. [file Image_3.jpeg]

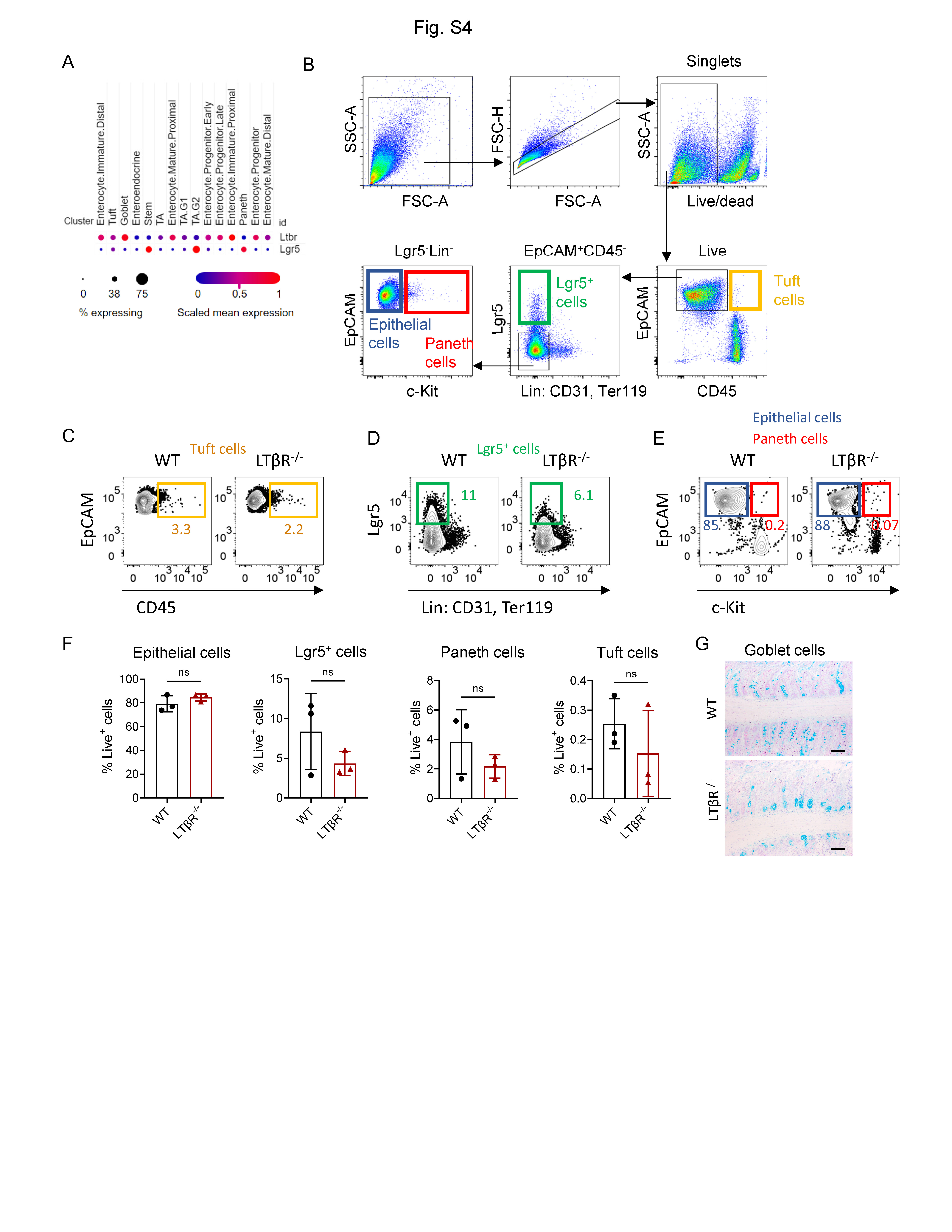

Supplement: Supplementary Figure 4 — Analysis of epithelial cell populations in the ileum of WT and LTβR-/- mice. (A) Expression of Ltbr and Lgr5 in various small intestine derived cell types was determined by single-cell RNA-seq. Data was obtained from the study conducted by Haber et al. (72), using the Broad Institute Single-Cell Portal for data analysis (https://portals.broadinstitute.org/single_cell/study/small-intestinal-epithelium). (B) WT and LTβR-/- mice were crossed with Lgr5-GFP reporter mice. GFP expression was induced by tamoxifen administration and mice were treated with MTX as in Figure 1A. Mice were euthanized on day 5 and ileum epithelial cells analyzed by flow cytometry. Gating strategy. Tuft cells: EpCAM+CD45+; Lgr5+ cells: EpCAM+Lgr5+CD31-Ter119-CD45-; Paneth cells: EpCAM+c-Kit+CD31-Ter119-CD45-; Epithelial cells: EpCAM+CD31-Ter119-CD45-. (C–F) Representative flow plot and frequency of cell populations. (G) Goblet cells analysis by Alcian Blue staining in small intestine. Scale bars, 100μm. Data show 1 of 2 independent experiments with similar results (n=3–5 per group). Data shown as mean ± SEM. Statistics were determined using unpaired t test. ns, not significant. [file Image_4.jpeg]

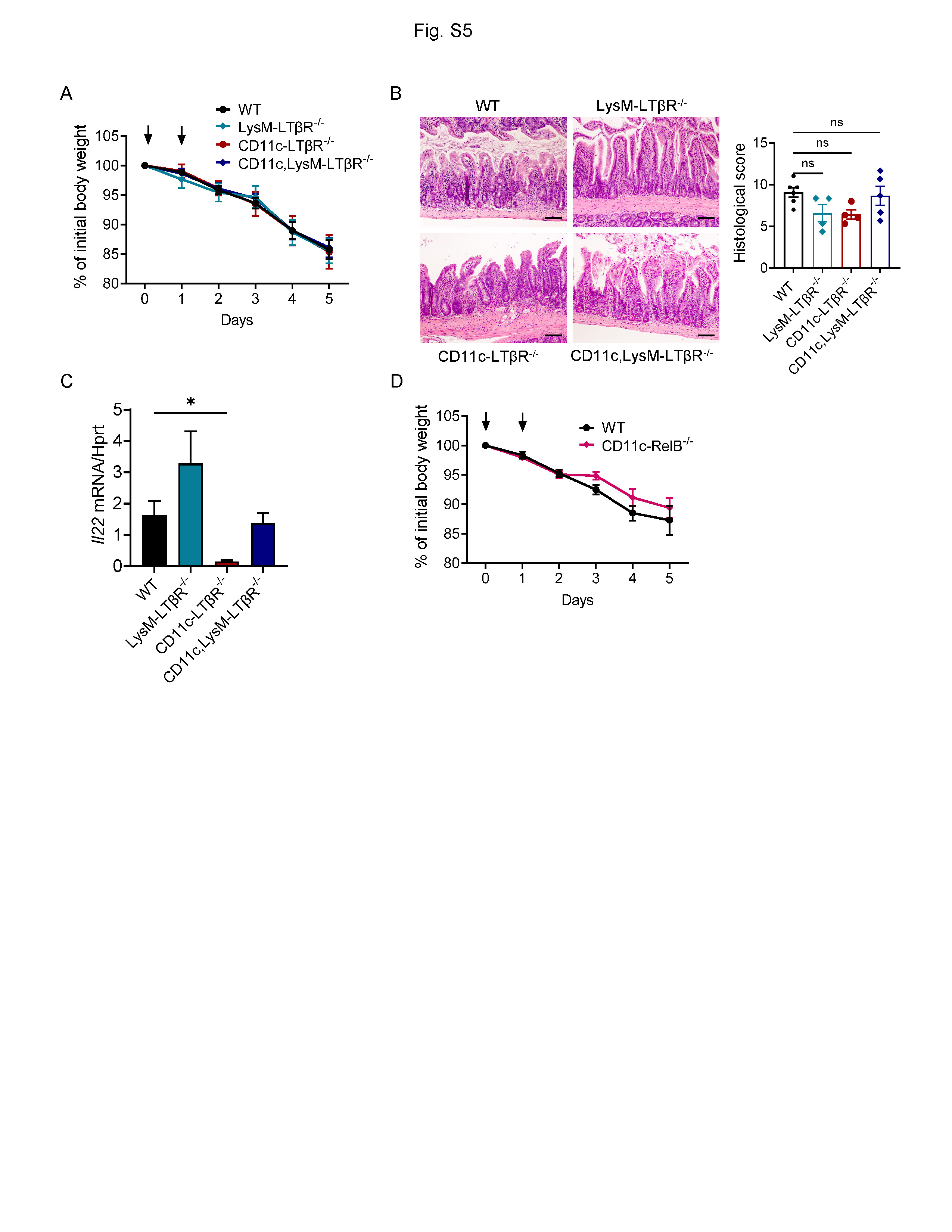

Supplement: Supplementary Figure 5 — LTβR signaling in macrophages and DCs is not essential for the protection from MTX induced intestinal injury. (A–D). WT, LysM-LTβR-/-, CD11c-LTβR-/-, CD11c, LysM-LTβR-/- and CD11c-RelB-/- mice were treated with MTX as in Figure 1A. (A, D) Body weight loss (n=9–14 mice per group) and (B) Representative H&E images (scale bars, 100μm) with histopathology scores. (C) IL-22 expression in the ileum on day 5 after MTX treatment. n= 5–8 mice per group. Data are combined from 3–5 independent experiments with similar results. Data shown as mean ± SEM. Statistics were determined using two-way ANOVA with Geisser-Greenhouse correction (A, D), Kruskal-Wallis test (B), unpaired t test (C). ns, not significant, * p<0.05. [file Image_5.jpg]
