# Supplementary material for: LTβR-RelB signaling in intestinal epithelial cells protects from chemotherapy-induced mucosal damage
Source: Front Immunol. 2024 May 30;15:1388496. doi: 10.3389/fimmu.2024.1388496 (PMC11169669; doi:10.3389/fimmu.2024.1388496)
Supplement: Supplementary file 6 [file DataSheet_1.pdf]

Supplementary Table 1. Primers for quantitative PCR

Gene	Forward primer	Reverse primer
Hprt	CTGGTGAAAAGGACCTCTCGAAG	CCAGTTTCACTAATGACACAAACG
Tnf	ACGGCATGGATCTCAAAGAC	AGATAGCAAATCGGCTGACG
Il6	ACAAGTCGGAGGCTTAATTACACAT	AATCAGAATTGCCATTGCACAA
Il1β	TGGACCTTCCAGGATGAGGACA	GTTCATCTCGGAGCCTGTAGTG
Ifng	TCAAGTGGCATAGATGTGGAAGAA	TGGCTCTGCAGGATTTTCATG
Il22	TCCGAGGAGTCAGTGCTAAA	AGAACGTCTTCCAGGGTGAA
Reg3β	ATGGCTCCTACTGCTATGCC	GTGTCCTCCAGGCCTCTTT
Reg3γ	ATGGCTCCTATTGCTATGCC	GATGTCCTGAGGGCCTCTT
Muc2	ACATCACCTGTCCCGACTTC	GAGCAAGGGACTCTGGTCTG
Cxcl1	GCTGGGATTCACCTCAAGAA	TGGGGACACCTTTTAGCATC
Cxcl2	CCTGGTTCAGAAAATCATCCA	CTTCCGTTGAGGGACAGC
Cxcl9	TGTGGAGTTCGAGGAACCCT	TGCCTCGGCTGGTGCTG
Cxcl10	TGAATCCGGAATCTAAGACCATCAA	AGGACTAGCCATCCACTGGGTAAAG
Cxcl13	CAGAATGAGGCTCAGCACAGC	CAGAATACCGTGGCCTGGAG
Ccl2	GCTGGAGCATCCACGTGTT	ATCTTGCTGGTGAATGAGTAGCA
LIGHT	GGAGACATAGTAGCTCATCTGCC	CCACCAATACCTATCAAGCTGGC
LTβ	CCTGTTGTTGGCAGTGCCTATC	GACGGTTTGCTGTCATCCAGTC
LTα	TCCACTCCCTCAGAAGCACT	AGAGAAGCCATGTCGGAG AA
NFkB2	TTGTCTGACATGGGTCTCCA	TGTCTTCTTTCACCTCTGCTGT
Mki67	GAGGAGAAACGCCAACCAAGAG	TTTGTCCTCGGTGGCGTTATCC
